# Pupil-linked arousal, cortical activity, and cognition in Alzheimer’s disease

**DOI:** 10.1093/braincomms/fcaf236

**Published:** 2025-07-22

**Authors:** Michael C B David, Emma-Jane Mallas, Lucia M Li, Magdalena A Kolanko, Ramin Nilforooshan, Man Lai Tsoi, Hanim Karakoc, Karen Hoang, Johanna Brandt, Charikleia Triantafyllou, Dragos C Gruia, Darije Custovic, Peter J Lally, Karl A Zimmerman, Care Research and Technology Group of the UK Dementia Research Institute, David Sharp, Danielle Wilson, Sarah Daniels, Ramin Nilforooshan, Matthew Harrison, Shlomi Haar, Mara Golemme, Payam Barnaghi, Paul Freemont, Ravi Vaidyanathan, Tim Constandinou, Gregory Scott, Derk-Jan Dijk, Pete Lally, Paresh Malhotra, Louise Robinson, Adam Hampshire, Michael David, Martina Del Giovane, Neil Graham, Magdalena Kolanko, Helen Lai, Lucia M Li, Mark Crook Rumsey, Emma Jane Mallas, Alina-Irina Serban, Eyal Soreq, Abidemi Otaiku, Megan Parkinson, Thomas Parker, Success Fabusoro, Emily Beal, Alan Bannon, Danilo Mandic, Ziwei Chen, Charalambos Hadjipanayi, Ghena Hammour, Bryan Hsieh, Amir Nassibi, Adrien Rapeaux, Ian Williams, Maowen Yin, Niro Yogendra, Maria Lima, Ting Su, Melanie Jouaiti, Maitreyee Wairagkar, Carlos Sebastian Castillo, Panipat Wattansiri, Thomas Martineau, Mayue Shi, Tianbo Xu, Bo Xiao, Alejandro Valdunciel, Reneira Seeamber, Annika Guez, Zehao Liu, Saksham Dhawan, Nan Fletcher-Lloyd, Samaneh Kouchaki, Alexander Capstick, Chloe Walsh, Louise Rigny, Ruxandra Mihai, Marirena Bafaloukou, Jin Cui, Ann-Kathrin Schalkamp, Yu Chen, Tianyu Cui, Nivedita Bijlani, Michael Crone, Kirsten Jensen, Martin Tran, Thomas Adam, Raphaella Jackson, Alexander Webb, Anne C Skeldon, Kevin Wells, Ullrich Bartsch, Ciro Della Monica, Kiran K G Ravindran, Damion Lambert, Sara Mohammadi Mahvash, Thalia Rodriguez Garcia, Victoria L Revell, Giuseppe Atzori, Lucinda Grainger, Hana Hassanin, James Woolley, Iris Wood-Campar, Janetta Rexha, Sarmad Al Gawwam, Subati Abulikemu, Julian Jeyasingh Jacob, Nathan Steadman, Federico Nardi, Cosima Graef, Alena Kutuzova, Assaf Touboul, Nicolas Calvo Peiro, Jenna Yun, Gaia Frigerio, Adela Desowska, Anastasia Gailly de Taurines, Ruxandra Mihai, Nina Moutonnet, Matthew Harrison, Sophie Horrocks, Brian Quan, Mark Woodbridge, Anna Joffe, Amer Marzuki, Ramsheed Abdul Rahim, Ramin Nilforooshan, Jessica True, Olga Balazikova, Nicole Whitethread, Matthew Purnell, Vaiva Zarombaite, Lucy Copps, Olivia Knight, Gaganpreet Bangar, Sumit Dey, Chelsea Mukonda, Jessica Hine, Luke Mallon, Saijal Jhala, Oliver Sargentoni, Amy Alves, Mahan Heydari, David Wingfield, Claire Norman, Anesha Patel, Ruby Lyall, Sanara Raza, Naomi Hassim, Pippa Kirby, John Patterson, Mike Law, Andy Kenny, Paresh A Malhotra, Gregory Scott, David J Sharp

**Affiliations:** UK Dementia Research Institute Care Research and Technology Centre, Imperial College London, London W12 0BZ, UK; Imperial College London, Brain Sciences, South Kensington, London SW7 2AZ, UK; UK Dementia Research Institute Care Research and Technology Centre, Imperial College London, London W12 0BZ, UK; Imperial College London, Brain Sciences, South Kensington, London SW7 2AZ, UK; UK Dementia Research Institute Care Research and Technology Centre, Imperial College London, London W12 0BZ, UK; Imperial College London, Brain Sciences, South Kensington, London SW7 2AZ, UK; UK Dementia Research Institute Care Research and Technology Centre, Imperial College London, London W12 0BZ, UK; Imperial College London, Brain Sciences, South Kensington, London SW7 2AZ, UK; UK Dementia Research Institute Care Research and Technology Centre, Imperial College London, London W12 0BZ, UK; Surrey and Borders Partnership NHS Foundation Trust, Leatherhead, Surrey KT22 7AD, UK; Imperial College London, Brain Sciences, South Kensington, London SW7 2AZ, UK; Imperial College London, Brain Sciences, South Kensington, London SW7 2AZ, UK; Imperial College London, Brain Sciences, South Kensington, London SW7 2AZ, UK; Imperial College London, Brain Sciences, South Kensington, London SW7 2AZ, UK; Imperial College London, Brain Sciences, South Kensington, London SW7 2AZ, UK; UK Dementia Research Institute Care Research and Technology Centre, Imperial College London, London W12 0BZ, UK; Imperial College London, Brain Sciences, South Kensington, London SW7 2AZ, UK; UK Dementia Research Institute Care Research and Technology Centre, Imperial College London, London W12 0BZ, UK; Imperial College London, Brain Sciences, South Kensington, London SW7 2AZ, UK; UK Dementia Research Institute Care Research and Technology Centre, Imperial College London, London W12 0BZ, UK; Imperial College London, Brain Sciences, South Kensington, London SW7 2AZ, UK; UK Dementia Research Institute Care Research and Technology Centre, Imperial College London, London W12 0BZ, UK; Imperial College London, Brain Sciences, South Kensington, London SW7 2AZ, UK; UK Dementia Research Institute Care Research and Technology Centre, Imperial College London, London W12 0BZ, UK; Imperial College London, Brain Sciences, South Kensington, London SW7 2AZ, UK; UK Dementia Research Institute Care Research and Technology Centre, Imperial College London, London W12 0BZ, UK; Imperial College London, Brain Sciences, South Kensington, London SW7 2AZ, UK; UK Dementia Research Institute Care Research and Technology Centre, Imperial College London, London W12 0BZ, UK; Imperial College London, Brain Sciences, South Kensington, London SW7 2AZ, UK

**Keywords:** noradrenaline, pupils, attention, neuromodulation, oddball

## Abstract

Arousal dysfunction contributes to impairments seen in Alzheimer’s disease. However, the nature and degree of this dysfunction have not been studied in detail. We investigated changes in tonic and phasic arousal using simultaneous pupillometry-EEG, relating these changes to locus coeruleus integrity, a key arousal nucleus. Forty Alzheimer’s disease participants and 30 controls underwent neuropsychological testing using the Alzheimer’s Disease Assessment Scale–Cognitive Subscale (ADAS-Cog), MRI designed to show contrast in the locus coeruleus as a measure of integrity and simultaneous pupillometry-EEG during 5 min of eyes-open resting-state. Pupillometry-EEG was then also applied during an oddball task which included a passive session and sessions in which responses to target stimuli were required, to test the effect of salience. Alzheimer’s disease had lower locus coeruleus integrity (*b* = −0.26, *P* = 0.02) and lower peak alpha frequency (tonic arousal) (*b* = −1.09, *P* < 0.001). Both were related to ADAS-Cog. There was a very strong relationship between pupil size and both periodic and aperiodic EEG power. Cortical slowing in Alzheimer’s disease affected this relationship, particularly at low frequencies. During the attentionally demanding oddball task, Alzheimer’s disease participants’ behavioural performance was impaired, with reduced accuracy and slower and more variable reaction times. They also had reduced pupil responses to salient stimuli (phasic arousal) (estimate = −0.19, *P* < 0.001). EEG and pupil measures of pre-stimulus tonic arousal were strongly correlated and predicted behavioural responses in both groups. Arousal fluctuations at rest and in response to stimuli are abnormal in Alzheimer’s disease as measured by combined pupillometry and EEG. Salient stimuli that require a behavioural response are accompanied by a phasic increase in arousal, demonstrated by pupil dilation to oddball stimuli. This response is slower and of smaller magnitude in Alzheimer’s disease patients. Cortical slowing (reduced peak alpha frequency) is seen in Alzheimer’s disease, and this is modulated by arousal level and relates to overall cognition. Pupil-linked arousal responses and alpha EEG fluctuations are tightly coupled, but cortical slowing in Alzheimer’s disease influences this coupling. The tools used here to measure neurophysiological arousal level have potential in understanding the nature of arousal system dysfunction in Alzheimer’s disease at the group level. These tools may also be used as biomarkers at the individual level in order to target patients most likely to benefit from arousal-modulating medications.

## Teaser

Combining pupillometry, EEG and MRI, David *et al*. report abnormalities in tonic and phasic arousal in Alzheimer’s disease. Locus coeruleus integrity, peak alpha frequency and pupil responses to oddballs were reduced. Cortical slowing affected the pupil–brain relationship. These results help characterize the effect of arousal dysfunction in relation to cognition.

## Graphical Abstract

**Graphical Abstract:**
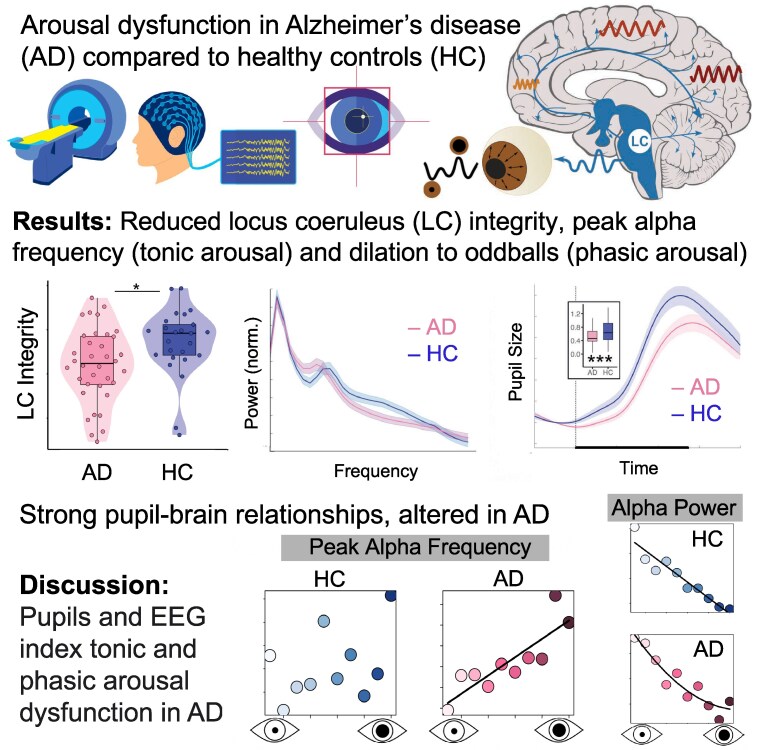

## Introduction

A system of ascending projections from brainstem nuclei, in conjunction with the cortico-thalamic system, determine ‘arousal’.^1,2^ Arousal dictates responsiveness to stimuli—environmental or internal.^3,4^ Arousal system dysfunction in Alzheimer’s disease (AD) has been described in various contexts including attention,^5^ the sleep–wake cycle,^6^behavioural regulation,^7,8^ and consciousness.^9^ A healthy arousal system is required for optimal cognitive performance, particularly attention and memory—core deficits in AD.^10,11^ However, the impact of changing arousal on cognition in AD is not well characterized.

Activity in the locus coeruleus (LC)—the brain’s principal source of noradrenaline—is key in determining arousal level.^11-13^ In AD, early tau pathology in the LC followed by cell death produces arousal system dysfunction, relating to disease progression and cognitive symptoms.^14,15^ In preclinical AD, both LC structural integrity and functional connectivity to the temporal lobe relate to pathological progression and cognitive decline.^16,17^ Reduced LC volume relates to impaired executive abilities early in AD.^18^ Also, there is evidence that noradrenergic medications improve cognition in AD.^19^ However, there remains a need to study fluctuations in arousal, which can be done using pupillometry and EEG.^20,21^

**Figure 1:**
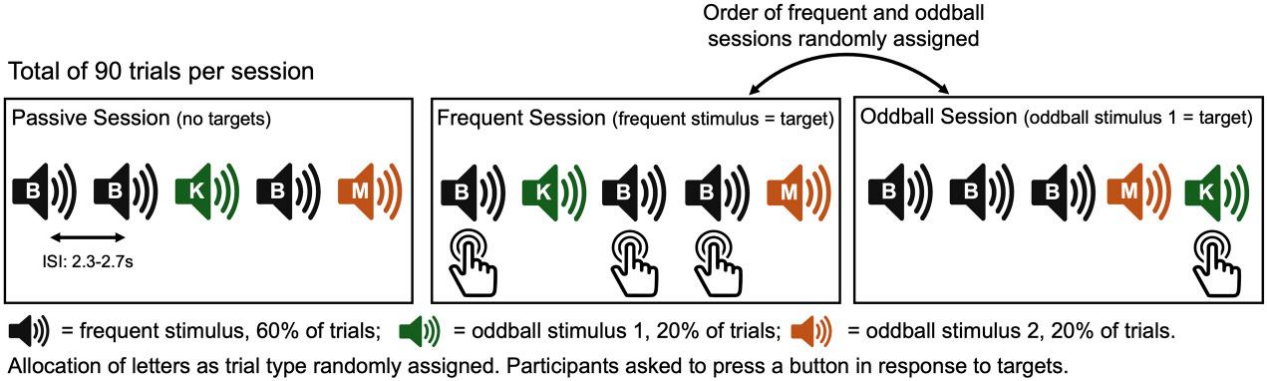
**Oddball task paradigm.** Schematic detailing the oddball task paradigm, consisting of three sessions: ‘passive’, ‘frequent’ and ‘oddball’. ISI, inter-stimulus interval.

Pupil size closely reflects activity in brainstem nuclei, including the LC’s tonic and phasic modes.^22-25^ The tonic mode represents basal activity level, and spontaneous pupil size fluctuations during wakeful rest provide a proxy measure.^22^ In the phasic mode, burst-like activity induces focused attention^12,26^ and can be studied using pupillary responses during tasks.^27-30^ Oddball tasks involve the presentation of infrequent target stimuli in a sequence of frequent distractors (Fig. 1). Stimuli induce arousal due to prediction error (infrequency) and salience (targets),^31,32^ although examples of its use in AD are limited.^33,34^

EEG alpha rhythms are modulated by noradrenergic projections to the thalamus.^35,36^ Different states of arousal have long been associated with distinct EEG features, including the frequency at which the alpha peak occurs.^21^ In AD, a general EEG ‘slowing’ is seen, demonstrated by a lower peak alpha frequency (in health, usually ∼10 Hz).^37^ In healthy aging and AD, this lower alpha peak relates to poorer global cognition and memory.^38,39^ Via the thalamus, the ascending arousal system suppresses alpha power when a task demands externally directed attention.^35,36,40^ Therefore, dysfunctional arousal-related control of alpha rhythms in AD affects cognition.^36^ However, arousal-related changes to alpha do not happen in isolation. Rather, changes in alpha are often accompanied by alteration in other frequency bands, including a shift in the distribution of power to the higher frequencies.^36^ Therefore, we explored the relationship between pupil-linked arousal and power in the delta, alpha and beta bands.^36,41^ In addition to oscillatory EEG activity, the aperiodic component, once disregarded as noise, is increasingly considered a source of meaningful signal.^42,43^ Changes in the aperiodic power distribution, the 1/f component, reflect arousal^44^ and relate to cognition.^42,45^ Together, evidence points to sub-cortical and cortical arousal dysfunctions as contributors to cognitive and neuropsychiatric symptoms in AD.^15,46^ Animal neurophysiology and studies in healthy individuals have consistently demonstrated pupil size and aspects of EEG probe a common arousal phenomenon related to these structures.^35,41^

We conducted a multi-modal investigation of the structure and function of the arousal system in AD. Using MRI, we measured LC contrast to index structural integrity,^47^ consistently reduced in AD.^48,49^ Using simultaneous pupillometry and EEG as arousal biomarkers, we tested the following hypotheses: (i) Arousal-related fluctuations in pupil size and cortical dynamics will strongly correlate during rest. (ii) Peak alpha frequency during rest, as a measure of cortical slowing driven by tonic arousal, will be reduced in AD. (iii) The correlations between pupil size and EEG power will be affected by the AD cortical slowing. (iv) Phasic arousal activity, measured by pupil dilation responses to oddball stimuli, will be reduced in AD. (v) Peak alpha frequency and pupil dilation responses will relate to Alzheimer’s Disease Assessment Scale–Cognitive Subscale (ADAS-Cog) as a measure of global cognition.

## Materials and methods

### Study design and participants

A total of 40 participants with AD were recruited as part of two studies: (i) ‘Minder’, an ongoing longitudinal community-based cohort study of people with dementia run by the Care, Research and Technology Centre of the UK Dementia Research Institute,^50^ and (ii) ‘Physiological Correlates of Noradrenergic Add-on Therapy’ (PCNorAD), an experimental medicine add-on study investigating noradrenergic dysfunction in patients with AD who have taken part in the ‘NorAD’ clinical trial.^51^ Participants recruited from ‘NorAD’ underwent the procedures for this study at least one month after completing the clinical trial, except one who entered ‘NorAD’ after this study. Thirty-two age-matched healthy controls (HCs) were also recruited as part of ‘PCNorAD’. The study involved participants attending for separate, 1-day visits: one for the resting-state and oddball task pupillometry-EEG assessment and one for MRI. The maximum time between MRI and pupillometry-EEG was 12 months (mean = 3 months).

AD participants all had a pre-existing clinical diagnosis of AD but were also discussed within a multidisciplinary team meeting, comprised of neurologists, psychiatrists and neuroradiologists, as part of this study. Diagnosis was based upon neuroimaging and cognitive assessments conducted for research, in addition to clinical history and investigations. On the EEG and MRI days of study, participants were asked to not consume caffeine, and drugs with noradrenergic action were withheld, but otherwise normal treatment was unaltered. HCs were included if >60 years old and with no symptoms of cognitive impairment. All participants were free of major neurological or psychiatric illnesses other than AD, and hence, one HC was excluded due to evidence of traumatic diffuse axonal injury on MRI. Cognitive profile was assessed using the ADAS-Cog 14^52^ within 3 months of pupillometry-EEG. One HC was excluded due to objective cognitive impairment defined as an ADAS-Cog score of >20. This gave a total of 40 AD and 30 HCs in the study. Two of the 40 AD participants did not undergo MRI, one due to severity of cognitive impairment and another due to claustrophobia. Four HCs declined an MRI.

### Ethical approval

The ‘Minder’ study was approved by the Health Research Authority’s London-Surrey Borders Research Ethics Committee (19/LO/0102). The ‘PCNorAD’ study was approved by the Health Research Authority’s London-Central Research Ethics Committee (18/LO/0249). All participants with capacity to consent provided written informed consent for participation and for their data to be included in publications. Those without capacity were enrolled on recommendation of an assigned consultee.

### MRI acquisition

MRI data were obtained using a Siemens Verio 3T MRI scanner with a 32-channel head coil. Participants underwent a T_1_-weighted 3D magnetization-prepared rapid acquisition gradient-echo (3D-MPRAGE) sequence and a 3D T_2_*-weighted multi-echo gradient-echo with magnetization transfer preparation pulse sequence designed to show LC contrast. Full parameters are found in Supplementary Methods.

### Pupillometry-EEG assessment

#### Set-up

Participants underwent simultaneous pupillometry and EEG during 5 min of eyes-open resting-state and then during an oddball task, conducted in a windowless room, shielded with a Faraday cage. Paradigms were run using MATLAB R2021a (MathWorks). High-density EEG was recorded continuously using a 256-channel hydrocel geodesic sensor net [Electrical Geodesics, Inc. (EGI)], following sizing and soaking of the cap in potassium chloride solution as per the manufacturer’s instructions. The pupil diameter (from here on referred to as size) was measured using an EyeLink 1000 Plus Desktop Mount eye-tracker. Full details of the set-up are in Supplementary Methods.

#### Resting-state and oddball paradigms

During the resting-state, participants were instructed to keep their eyes open and stay awake, stare at a central fixation cross and ‘let their mind wander and think of nothing in particular’. A researcher was present in the room to ensure they did not fall asleep. The oddball task involved participants listening to a sequence of auditory stimuli, consisting of spoken letters B, K and M played through the monitor speaker. The task paradigm (Fig. 1) was divided into three ‘sessions’, with full details in Supplementary Methods. One letter was played for 60% of trials (‘frequent’) and the other two letters were played for 20% of trials each (‘oddballs’). The first session was the ‘passive session’, in which participants simply listened to the (‘non-target’) stimuli. For the second and third sessions, participants were asked to respond to an assigned letter (‘targets’) by pressing a button, and not others (‘non-targets’). For one of these two sessions, the target stimuli were frequently occurring (‘frequent session’), and for the other, they were one of the two oddball stimuli (‘oddball session’).

### Data pre-processing

#### EEG data

Raw EEG data were pre-processed, cleaned and quality assessed using the Harvard Automated Processing Pipeline for EEG (HAPPE) version 2^53^ which utilizes EEGLAB functions and was implemented within MATLAB. This pipeline has been shown to perform better than manual editing in clinical populations.^53^ Following pre-processing, one participant’s oddball data was excluded as event labels were not saved due to a technical error, and two AD participants were excluded from resting state EEG analysis due to excess artefact resulting in all segments being rejected. Details of pre-processing steps and parameters can be found in Supplementary Methods.

#### Pupil data

Pupil data were visually inspected for excessive missing data including excessive/poorly interpolated blinks (below), and nine participants were excluded from resting state analysis (one of whom was also excluded for poor EEG data), and three for the oddball, on this basis (see Supplementary Fig. 1 for examples). Pupil data from the resting-state and oddball were pre-processed using the same in-house pipeline, details of which are in Supplementary Methods.

### Data analysis

#### Locus coeruleus contrast quantification

We used an adaptation of the method in Clewett *et al.*^54^ with full details in the Supplementary Methods. Extending the previously reported method, we also examined two slices above and below the middle LC slice, giving a total of five slices.^54^ The contrast ratio in the LC was measured as a ratio compared to the reference regions in the nearby pons for each slice separately calculated using the following equation: (mean of left and right LC contrast − mean reference contrast)/standard deviation of reference contrast.^55^ Then, this contrast-to-noise ratio was averaged for the five slices, yielding a single overall contrast measure. Two independent raters evaluated each scan. The ICC between the two raters was high (0.88–0.96). Where there was a large disagreement of contrast ratio between raters (>0.45) (*N* = 5), scans were re-examined, and a consensus was reached between raters. Two AD LC contrasts could not be quantified due to image artefact and one HC contrast was excluded as an outlier (>1.5 × IQR above the third quartile of all participants).

#### Resting-state pupillometry and EEG

Skewness and kurtosis of pupil data and the number of spontaneous pupil peaks/troughs was computed. These were identified using the ‘findpeaks’ MATLAB function with a minimum prominence of 0 and a minimum distance between peaks of 3 s.^56^ Pupil time course ±3 s around peaks/troughs was averaged for AD and controls.

The alpha frequency band was defined as 6–10 Hz. This is lower than typical ranges (∼8–12 Hz), but was used based on literature showing the peak in power representative of the alpha band is typically seen at lower frequencies in healthy aging and in AD. Also, analysis of the average peak alpha frequency for each group [AD = 7.42 Hz (1.18), HC = 8.60 Hz (0.98), mean (SD)] showed that a range of 6–10 Hz was reasonable to capture the alpha activity for participants in both groups equally. The resting-state power spectrum was calculated using a fast Fourier transform, and power was extracted from the alpha band. Peak alpha frequency was calculated using the MATLAB ‘restingIAF’ function,^57^ specifically the centre of gravity metric (full details of parameters used is reported in Supplementary Methods). The same method was used to identify pupil peaks/troughs was applied to the alpha time course.

As well as alpha (6–10 Hz), we analysed data in the delta (1–4 Hz) and beta (12–30 Hz) bands. For each channel and frequency band of interest, the EEG power time course was extracted from the output of a multitaper time-frequency transformation performed using the ‘ft_freqanalysis’ FieldTrip function.^58^ For each 20 s epoch, the pupil time course was extracted and cross-correlations computed between each band-power time course and lagged versions of the pupil time course, for a range of lags between −3 and 3 s in increments of 0.1 s (see below).

The temporal relationship between cortical activity and pupil size is complex. Animal work measuring the delay between direct LC stimulation and pupil dilation puts it between 0.3 and 0.91 s.^23,59^ External stimuli, processed by the cortex, evoke pupil dilations via top-down mechanisms, which peak at a delay of about 1 s, including in AD.^33,60^ Arousal fluctuations occur spontaneously at rest and in response to external stimuli. Both of which induce cortical changes, which are followed by LC changes.^41,60^ The neurological and muscular delay inherent in this pathway from the cortex to the LC to the pupils leads to pupil changes lagging behind the cortex.^23^ This is reflected in our own data where the greater beta values (negative and positive) are all seen in the 0 to −2 s lag periods. This is very clear in Supplementary Fig. 2C–F which shows that spikes in activity occur in the cortex before the pupils. Therefore, we chose to focus our investigation on the relationship between the pupils and the EEG power with and without a 1 and 2 s shift to capture the full range of significant correlations (i.e. moving the pupils forward in time, to account for them lagging behind the cortex. This is referred to below as a −1 and −2 s lag, respectively).

To further measure how power in different bands changes as a function of pupil size, the pupil data were binned at the participant level into 10 deciles (bins) and power values for each band were averaged to yield a single power value for each bin, for each participant, and then, the power values for each bin were averaged across participants in each group.

For analysis of peak alpha frequency as a function of pupil size, the pupil and EEG data were first divided into 2 s epochs. Then, the pupil size was averaged for each epoch and each epoch designating into one of 10 bins based on average pupil size, for later use in the peak alpha frequency analysis and ‘FOOOF’ analysis (below). The ‘restingIAF’ function^57^ was applied to the EEG segments that had been assigned to each bin, for each participant. The resulting peak alpha frequency values for each bin were then averaged across participants, for each group, as above.

To separate out the aperiodic component of the power spectra, we used the FieldTrip wrapper for the MATLAB implementation of ‘FOOOF’ in the Brainstorm toolbox.^42^ Details of the parameters used are included in Supplementary Methods. The function was applied to the power spectra of each individual’s entire resting-state time course. Separately, it was also applied to the averaged spectra calculated for the binned 2 s epochs, as for the peak alpha frequency analysis.

#### Oddball task analyses

Accuracy was calculated as the percentage of correct trials out of the 180 trials in the latter two sessions (in which responses were required). Only correct trials were used for further analysis. Reaction time was recorded for each correct target trial and used to calculate mean and coefficient of variation (CoV) (standard deviation/mean) for each participant, as a measure of attentional state that is sensitive to AD.^41^

Trial-level time-frequency transformation (multitaper method) was performed. Power values were averaged over time and channels to derive a single alpha power value for the global baseline (0.5 s pre-stimulus) for each trial. The baseline power for each trial was then converted to relative power by dividing by the total power (1–80 Hz) for that trial baseline period.

For correct trials with no missing pupil data after blink interpolation, pupil data for each trial period (0.5 s pre-stimulus to 2 s post-stimulus) were baseline-normalized by subtracting the average pupil size during the 0.5 s pre-stimulus.^61^ The average of the baseline-corrected pupil size during the 2 s post-stimulus was taken as the pupil dilation response. The time taken to reach maximum pupil size (latency) was also recorded. Groups were compared using individuals’ average pupil dilation for each trial type. Group-average pupil time courses for each trial type were plotted, and points of difference in the time courses were identified.

For each participant separately, all correct trials were divided into quintiles based on baseline pupil size. We averaged the baseline alpha power for all trials in each quintile and then averaged across participants, for that quintile. Then, looking at only all correct target trials (oddball and frequent), we divided each participant’s trials into quintiles based on the baseline pupil size and then averaged the reaction time for each quintile, as above. Next, we did the same using baseline alpha power for correct target trials.

Then, we took only the correct oddball target trials (as these induced the greatest pupil dilation) and divided each participants’ trials into quintiles based on the baseline pupil size and then averaged the pupil dilation response for each quintile, as above. Finally, we did the same using baseline alpha power for correct oddball target trials.

### Statistical analysis

For analysis of relationships between LC contrast, ADAS-Cog, peak alpha frequency and alpha power (during resting-state), and for group differences in these measures and aperiodic exponents, linear regression models were used with age and sex included as covariates, as well as years of education when ADAS-Cog was the dependent variable. Relationships between ADAS-Cog score and other variables were not tested in the control group as this instrument is specifically designed to monitor changes in cognition in AD, and there was minimal variance in the scores amongst controls.

A linear mixed-effects model was employed to test for the effect of group and pupil deciles on EEG band power and the aperiodic exponent, as well as their interaction. The model included a random intercept for individual participants to account for repeated measures within each participant. For quintile-based analysis of pupil, EEG and reaction time measures in the oddball task, linear mixed-effects models were again used. For examination of group differences in pupil responses during the oddball task, linear mixed-effects were also used, with the pupil metric (dilation response/time to maximum) as the dependent variable and group, trial type and their interaction as fixed effects, again including a random intercept for participant. Full details on statistical approaches are reported in Supplementary Methods.

## Results

### Participant characteristics

In total, 40 AD participants and 30 HCs were included in the study, matched for age and sex (Table 1). The AD group had significantly fewer years of full-time education [*t*(54.95) = −2.59, *P* = 0.01]. Twenty-eight AD were on acetylcholinesterase inhibitors at the time of testing. The average number of years of dementia symptoms in the AD group was 5.5 (3.1) [mean (SD)]. The average ADAS-Cog score was significantly higher in AD [mean (SD) = 26.0 (16.6)] than HC [mean (SD) = 37.9 (11.8)] [*t*(52.64) = 13.63, *P* < 0.001].

**Table 1:** Participant characteristics

	HC	AD	Statistic	*P*
Total included in analyses	30	40		
Age, years, mean (SD)	76.7 (5.4)	75.5 (7.7)	*t* = −0.75	0.46
Sex, % female	50.0	45.0	*χ* ^2^ = 0.03	0.86
ADAS-Cog, mean (SD)	10.2 (4.4)	37.9 (11.8)	*t* = 13.63	<0.001
Full-time education, years, mean (SD)	15.1 (3.7)	13.0 (3.0)	*t* = −2.59	0.01
AChEI, drug, daily dose (*n*)		Don 5 mg (4), Don 10 mg (19), Gal 16 mg (3), Gal 24 mg (1), Riv 1.5 mg (1), nil (12)		
Dementia symptoms, years, mean (SD)		5.5 (3.1)		

*t*-test used to compare groups for continuous variables and chi-square for % female.

Don, donepezil; Gal, galantamine; Riv, rivastigmine; HC, healthy control; AD, Alzheimer’s disease; ADAS-Cog, Alzheimer’s Disease Assessment Scale–Cognitive Subscale; AChEI, acetylcholinesterase inhibitors.

Following exclusion for poor data quality and outliers (see Materials and methods), the number of subjects included in the analysis for each of the respective sections was as follows: resting state EEG: HC = 30, AD = 38; resting state EEG and pupillometry: HC = 28, AD = 33; oddball behavioural and EEG: HC = 30, AD = 39; oddball behavioural, EEG and pupillometry: HC = 29, AD = 37; and MRI: HC = 25, AD = 36.

### Locus coeruleus contrast is lower in Alzheimer’s disease and relates to cognition

**Figure 2:**
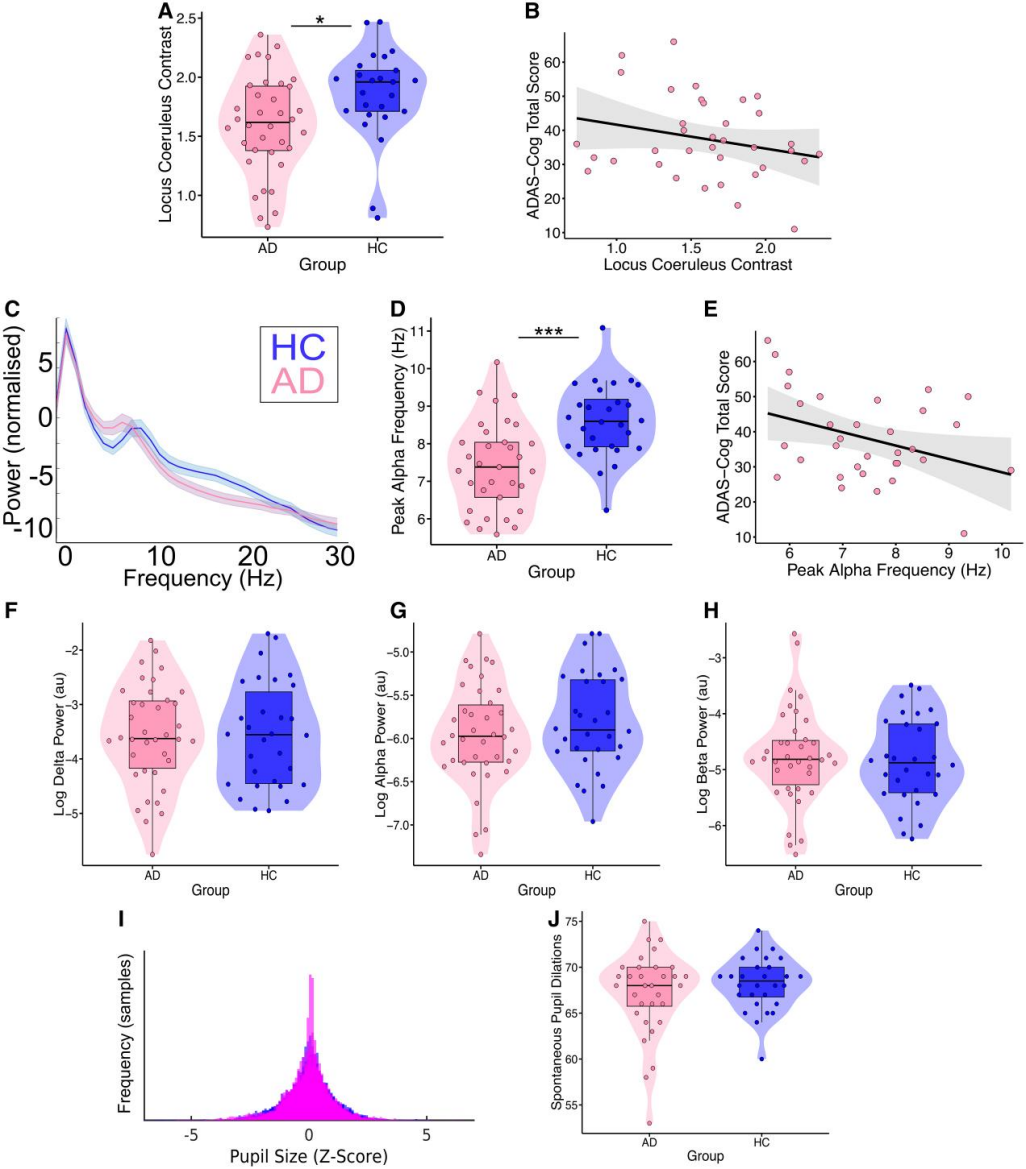
**Relationship between locus coeruleus contrast, EEG slowing and cognition in Alzheimer’s disease.** (**A**) Violin plot comparing locus coeruleus contrast between groups calculated as a ratio between the contrast in the locus coeruleus and a reference region in the pons (*b* = −0.26, *P* = 0.02). (**B**) Relationship between locus coeruleus contrast and ADAS-Cog total score (*b* = −0.01, *P* = 0.02). (**C**) Spectra of normalized power separated by group showing a shift to lower frequency of the alpha peak in AD. (**D**) Violin plots showing a reduced peak alpha frequency in AD (*b* = −1.09, *P* < 0.001). (**E**) Scatter plot of AD individuals’ peak alpha frequency against their ADAS-Cog score (*b* = −4.37, *P* = 0.03). (**F–H**) Violin plots comparing log power measured in arbitrary units (au), in the delta, alpha and beta bands, respectively, during the resting-state, between the two groups (all non-significant). (**I**) Histograms showing distribution of pupil size data for all individuals overlayed, demonstrating no difference in the shape of the distributions between subjects in the AD group (pink) and the control group (blue). (**J**) Violin plots showing no significant difference between groups in the number of spontaneous pupil dilations during resting-state. For **B**, **C** and **E**, shaded areas represent standard error of the mean. All statistics shown are results of linear regression including age and sex as covariates. Plotted line in scatter plots represents best fit through the raw data. HC, healthy control; AD, Alzheimer’s disease; ADAS-Cog, Alzheimer’s Disease Assessment Scale–Cognitive Subscale. *P < 0.05; ***P < 0.001.

AD participants had significantly lower contrast in the LC (*b* = −0.26, SE = 0.11, *t* = −2.45, *P* = 0.02) (Fig. 2A). There was a significant negative relationship between ADAS-Cog score and LC in the AD group (*b* = −0.01, SE = 0.01, *t* = −2.56, *P* = 0.02), indicating worse cognition with lower LC contrast (Fig. 2B).

### Lower peak alpha frequency, which relates to locus coeruleus contrast and cognition in Alzheimer’s disease

The AD group showed significantly reduced peak alpha frequency. During 5 min of eyes-open resting-state, there was a lower peak alpha frequency in AD participants [mean (SD) = 7.42 Hz (1.18)] compared to controls [mean (SD) = 8.60 Hz (0.98)] (*b* = −1.09, SE = 0.25, *t* = −4.30, *P* < 0.001) (Fig. 2D). Peak alpha frequency in the AD group was negatively related to cognitive function measured by ADAS-Cog score (worse cognition with lower peak alpha frequency) (*b* = −4.37, SE = 1.89, *t* = −2.32, *P* = 0.03) (Fig. 2E). There was no group difference in delta, alpha or beta power (*P* > 0.05) (Fig. 2F–H). On investigating the relationship between peak alpha frequency and LC contrast, there was a significant interaction of group (*b* = −1.21, SE = 0.52, *t* = −2.32, *P* = 0.02), due to a positive relationship in the AD group (*b* = 1.10, SE = 0.48, *t* = 2.28, *P* = 0.03) but no relationship in the control group (*P* > 0.05).

### Pupil size strongly predicts EEG power

We next investigated pupillary dynamics as a measure of arousal and their relationship to EEG. First, we compared the nature of pupil fluctuations themselves, between groups. Pupil size distribution and dilation/constriction events were similar between groups. The distribution of pupil sizes for each subject was comparable between groups in terms of skewness and kurtosis (*P* > 0.05) (Fig. 2I). In addition, the number of spontaneous local peaks/troughs in pupil size (dilation/constriction events) was not significantly different between groups (*P* > 0.05) (Fig. 2J).

**Figure 3:**
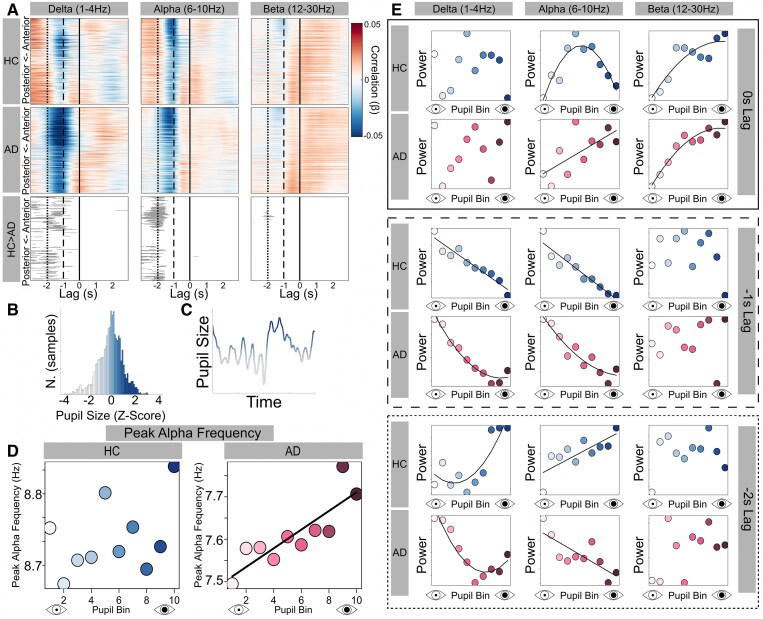
**Pupil size relates to EEG power and alpha frequency during resting-state.** (**A**) Pearson correlation values (β) with pupil size across all channels, sorted from anterior (top) to posterior (bottom), and for lags ranging from −3 to +3 s, averaged within three frequency bands of interest: delta (1–4 Hz) (left), alpha (6–10 Hz) (centre) and beta (12–30 Hz) (right), split by group (HC above, AD below). Negative lags are indicative of EEG preceding pupil, and vice versa. Positive correlations are depicted with red and negative with blue. Point of 0 s lag marked with solid vertical black line, −1 s lag with dashed line and −2 s lag with dotted line. Bottom row shows regions highlighted in grey where HC β > AD β (*P* < 0.05). (**B**) Example distribution of pupil data from one participant, arranged by size, binned into deciles (each decile in a different colour). (**C**) Representative snapshot of pupil time course with deciles indicated by colour. (**D**) Peak alpha frequency (centre of gravity), as a function of pupil size, as above, split by group (HC above in blue, AD below in pink). (**E**) *Z*-scored spectral power, averaged across the same three frequency bands, as a function of pupil size, split by group (HC above in blue, AD below in pink). Top plots surrounded by solid box represent point indicated with a solid line in **A** at time of 0 s lag between pupils and EEG power. Middle plots surrounded by dashed box represent point indicated with a dashed line in **A** at time of −1 s lag. Bottom plots surrounded by dotted box represent point indicated with a dotted line in **A** at time of −2 s lag. For **D** and **E**, the data points represent data for 10 deciles which is averaged at the decile level within each subject and then across subjects within each group. Adjusted *R*-squared and *P*-values for each correlation found in Supplementary Table 1 or 2. Significant correlations (*P* < 0.05) indicated with best fit line. Linear or quadratic line used according to that with lower Bayesian information criterion. AD, Alzheimer’s disease; HC, healthy control.

Next, we looked at fluctuations in pupil size in conjunction with cortical activity. There were strong relationships between pupillary responses and EEG dynamics that varied depending on the shift applied to the pupil time course (Fig. 3A). Pupil size was strongly negatively correlated with both delta and alpha power in both AD and controls at a lag of ∼−1 s (i.e. when EEG preceded pupils). In contrast, there were group differences in this relationship when analysed at a longer lag between the EEG and pupil time courses. The negative correlation extended to lags of between −1 and −2 s in the AD group, whereas by −2 s, a positive correlation was present in controls (Fig. 3A).

We further investigated the relationships between pupil size and EEG by binning pupil size into deciles and measuring against power (Fig. 3E; Supplementary Table 1). For alpha power, at 0 s lag, controls showed a significant negative quadratic relationship between pupil size and EEG power, whereas the AD group showed a significant positive linear relationship. For beta power, both groups showed a significant negative quadratic relationship. For alpha and delta power at −1 s lag, both groups showed a negative relationship, with the controls showing a linear relationship and AD a quadratic one. The relationship between pupil size and EEG power showed clear group differences when analysed with −2 s lag. In delta, the control group showed a significant up-sloping, positive quadratic relationship, whereas in AD, the preferred model was a down-sloping, negative quadratic. For alpha power, there was a significant positive relationship in the control group and a significant negative relationship in the AD group, with linear models preferred in both.

We then investigated if the relationship between pupils and EEG power between the two groups differed as a result of the cortical slowing seen in AD. The correlation (Pearson *R* value) between pupil size and delta/alpha power was calculated for each participant. For delta power with −2 s lag, linear regression with group as an interaction term showed a significant positive relationship between peak alpha frequency and *R* value, indicating that the greater the cortical slowing (lower peak alpha frequency), the more negative the relationship between pupil decile and delta power (*b* = 0.77, SE = 0.36, *t* = 2.12, *P* = 0.04). There was no significant group interaction and no relationship with *R* values from pupil size against alpha power.

To further investigate the fluctuations in pupil time course that occur at a lag relative to the spontaneous fluctuations in alpha power during rest, we plotted the group-level average pupil time course relative to peaks and troughs in the alpha time course (Supplementary Fig. 2E and F). This revealed a deflection in the pupil time course in the opposing direction to the alpha power, approximately 1 s after the alpha event, in both groups. The pupil response in the AD group was relatively sluggish, taking longer to peak and return to baseline, compared to the control group.

### Pupil size predicts peak alpha frequency in Alzheimer’s disease

We also tested the relationship between pupil decile and peak alpha frequency. At −1 s lag, in the AD group, there was a significant positive linear relationship between pupil decile and peak alpha frequency (*R*^2^ = 0.70, *P* = 0.002) that was not seen in the control group (*R*^2^ = 0.06, *P* > 0.05) (Fig. 3D). There was no significant relationship at 0 or −2 s lag, for either group (Supplementary Table 2).

### Exponent of aperiodic EEG decreases with increasing pupil-linked arousal

**Figure 4:**
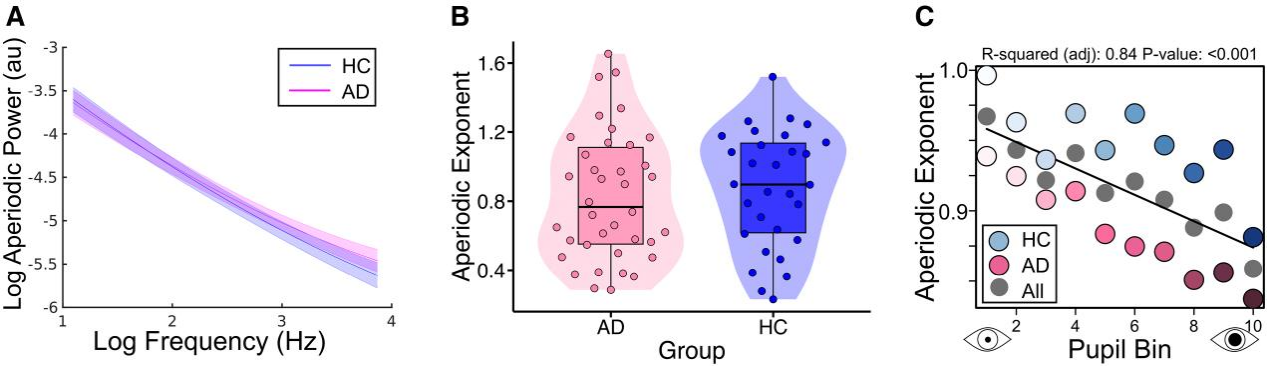
**Pupil size relates to aperiodic EEG power during resting-state.** (**A**) Spectra of log aperiodic power in arbitrary units (au) separated by group. (**B**) Violin plots showing the exponent of the aperiodic power spectra. There was no significant difference between groups when accounting for age and sex. (**C**) Exponent of aperiodic power as a function of pupil size (at −1 s lag), shown for all participants (grey) (*R*^2^ = 0.84, *P* < 0.001) and split by group [HC (blue) *R*^2^ = 0.51, *P* = 0.01; AD (pink): *R*^2^ = 0.96, *P* < 0.001]. The data points represent data for 10 deciles which is averaged at the decile level within each subject and then across subjects within each group. Best fit line, adjusted *R*-squared and *P*-values on plot refer to grey scatter points. AD, Alzheimer’s disease; HC, healthy control.

There was also a very strong relationship between pupillary dynamics and the aperiodic component of the EEG spectra. We separated out the aperiodic component of the EEG power spectra during the resting-state and measured the exponent of the resulting spectra for each individual (Fig. 4). There was a significant linear relationship between the aperiodic exponent and pupil decile across all subjects, with pupil data shifted forward 1 s (−1 s lag). A similar relationship was seen in both groups separately, although stronger in AD (all: adjusted *R*^2^ = 0.84, *P* < 0.001; AD: adjusted *R*^2^ = 0.96, *P* < 0.001; HC: adjusted *R*^2^ = 0.51, *P* < 0.01) (Fig. 4C). A linear mixed-effects model and subsequent ANOVA revealed a significant effect of pupil decile [F(9, 522) = 7.54, *P* < 0.001, η^2^ = 0.12], but despite a visible trend towards lower exponents in all pupil deciles in the AD group (Fig. 4C), there was no significant group effect and no group:pupil decile interaction (*P* > 0.05). At 0 s lag (all: adjusted *R*^2^ = 0.85, *P* < 0.001; AD: adjusted *R*^2^ = 0.77, *P* < 0.001; HC: adjusted *R*^2^ = 0.52, *P* = 0.01) and −2 s lag (all: adjusted *R*^2^ = 0.78, *P* < 0.001; AD: adjusted *R*^2^ = 0.66, *P* = 0.002; HC: adjusted *R*^2^ = 0.34, *P* = 0.05), there was a similar trend of significant linear negative relationship between pupil size and aperiodic exponent (Supplementary Fig. 3). There was no group difference in exponents (*P* > 0.05) (Fig. 4B).

### Pre-stimulus pupil size predicts dilation and behavioural responses, which are impaired in Alzheimer’s disease

AD participants showed impaired performance on the oddball task. Linear regression showed significantly shorter reaction times (*b* = −0.07, SE = 0.03, *t* = −2.41, *P* = 0.02), lesser variability (CoV) of reaction times (*b* = −0.10, SE = 0.03, *t* = −3.9, *P* < 0.001) and greater accuracy (*b* = 4.37, SE = 2.03, *t* = 2.16, *P* = 0.03) in the control group compared to AD. Worse ADAS-Cog score predicted slower reaction times in the AD group (*b* = 0.01, SE < 0.01, *t* = 2.38, *P* = 0.02), as well as worse accuracy (*b* = −0.28, SE = 0.13, *t* = −2.12 *P* = 0.04), but not CoV of reaction times (*P* > 0.05).

**Figure 5:**
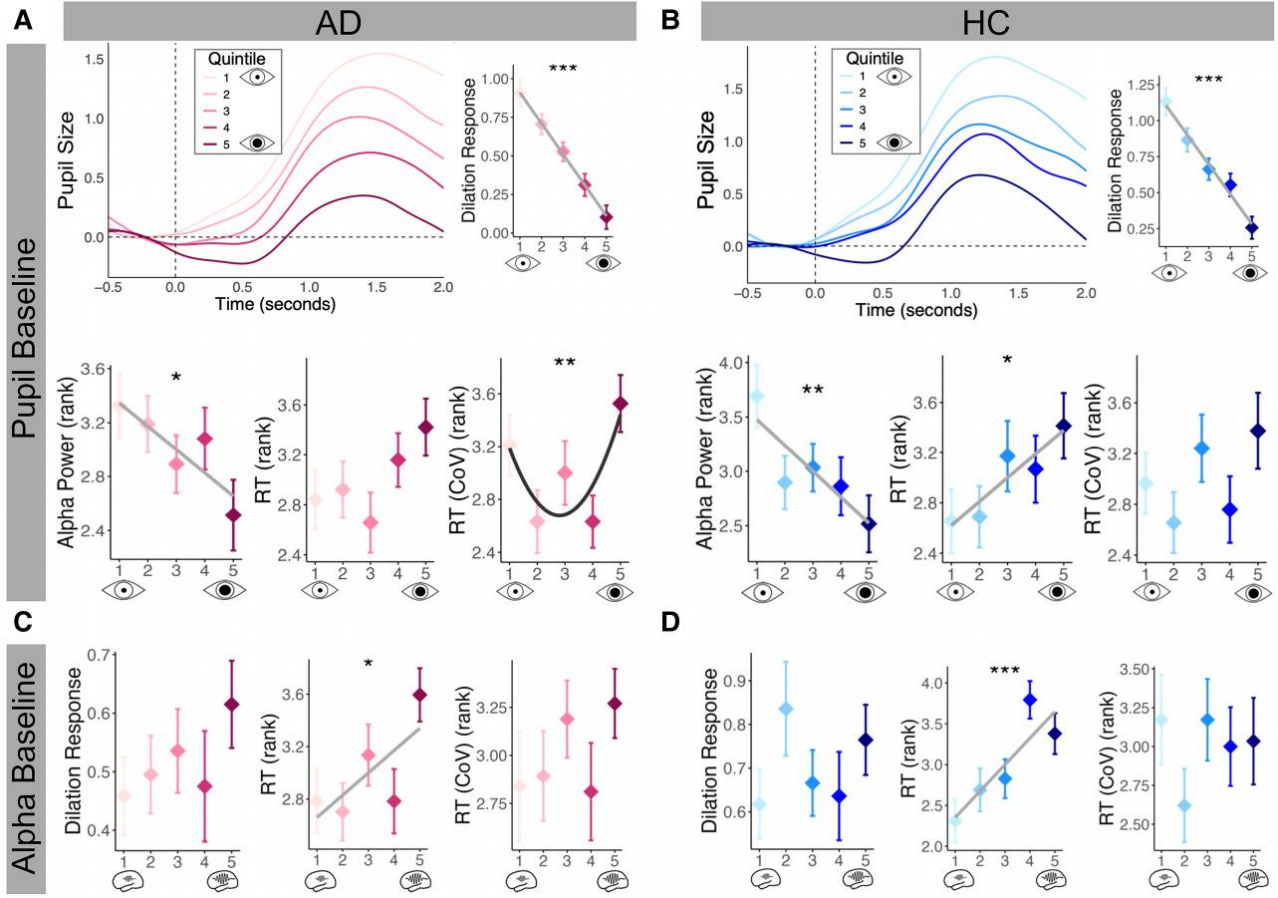
**Pre-stimulus pupil size and EEG power relates to pupil dilation response and reaction times.** (**A**) AD group (pink): top row shows pupil dilation responses to correct oddball target trials only, split into quintiles by baseline pupil size. Second row shows baseline (0.5 s pre-stimulus) relative alpha EEG power (rank converted) (left) (HC: *b* = −0.24, *P* = 0.004; AD: *b* = −0.17, *P* = 0.02), RTs (centre) (HC: *b* = 0.19, *P* = 0.02; AD *P* > 0.05) and CoV of RTs (right) (HC: *P* > 0.05; AD: *b* = 0.16, *P* = 0.01), both rank converted, as a function of baseline pupil size quintile, for all correct target trials (frequent and oddball). (**B**) As in **A** but for controls (blue). (**C**) AD group (pink) and (**D**) HC group (blue): pupil dilation responses (left) (HC and AD: *P* < 0.05), RT (HC: *b* = 0.32, *P* < 0.001; AD: *b* = 0.17, *P* = 0.02) and CoV of RTs (HC and AD: *P* < 0.05), as a function of baseline alpha power. Significant relationships (*P* < 0.05) indicated with best fit line; linear in light grey and quadratic in dark grey. *P < 0.05; **P < 0.01; ***P < 0.001. AD, Alzheimer’s disease; HC, healthy control; RT, reaction time; CoV, coefficient of variation.

We used simultaneous pupillometry and EEG during the task to directly measure the impact of arousal on behaviour. The pre-stimulus baseline period was used to measure tonic activity and attentional state, whilst the pupil responses to stimuli allowed the influence of phasic changes in arousal to be investigated. For both groups, there was a significant negative linear relationship between baseline pupil size and alpha power, in the pre-stimulus period (HC: estimate = −0.24, SE = 0.08, *t* = −2.93, *P* = 0.004; AD: estimate = −0.17, SE = 0.07, *t* = −2.38, *P* = 0.02) (Fig. 5A and B). Additionally, there was a strong negative relationship between baseline pupil size and pupil dilation response to oddball targets in both groups (HC: estimate = −0.21, SE = 0.02, *t* = −10.61, *P* < 0.001; AD: estimate = −0.20, SE = 0.02, *t* = −10.57, *P* < 0.001) (Fig. 5A and B). For target trials, in the control group, there was a significant positive relationship between baseline pupil size and reaction time (larger pupils, slower reaction times) (estimate = 0.19, SE = 0.08, *t* = 2.31, *P* = 0.02) (Fig. 5B), whereas in AD, there was a significant positive quadratic relationship between baseline pupil size and CoV of reaction time (estimate = 0.16, SE = 0.06, *t* = 2.61, *P* = 0.01), suggesting that the less variable reaction times—indicative of greater attentional engagement—occurred at an intermediary state of tonic arousal (Fig. 5A).

### Pre-stimulus alpha power predicts behavioural response but not pupil dilation response

Target trials were then binned into quintiles based on baseline pre-stimulus alpha power. In both groups, there was a significant positive relationship between baseline alpha power and reaction time (HC: estimate = 0.32, SE = 0.08, *t* = 4.10, *P* < 0.001; AD: estimate = 0.17, SE = 0.07, *t* = 2.34, *P* = 0.02), in keeping with the theory that suppressing alpha power is conducive for improved external attentional engagement (Fig. 5C and D).^30,40^ Baseline alpha power did not predict pupil dilation response (Fig. 5C and D).

### Pupil dilation response is impaired in Alzheimer’s disease and relates to cognition

**Figure 6:**
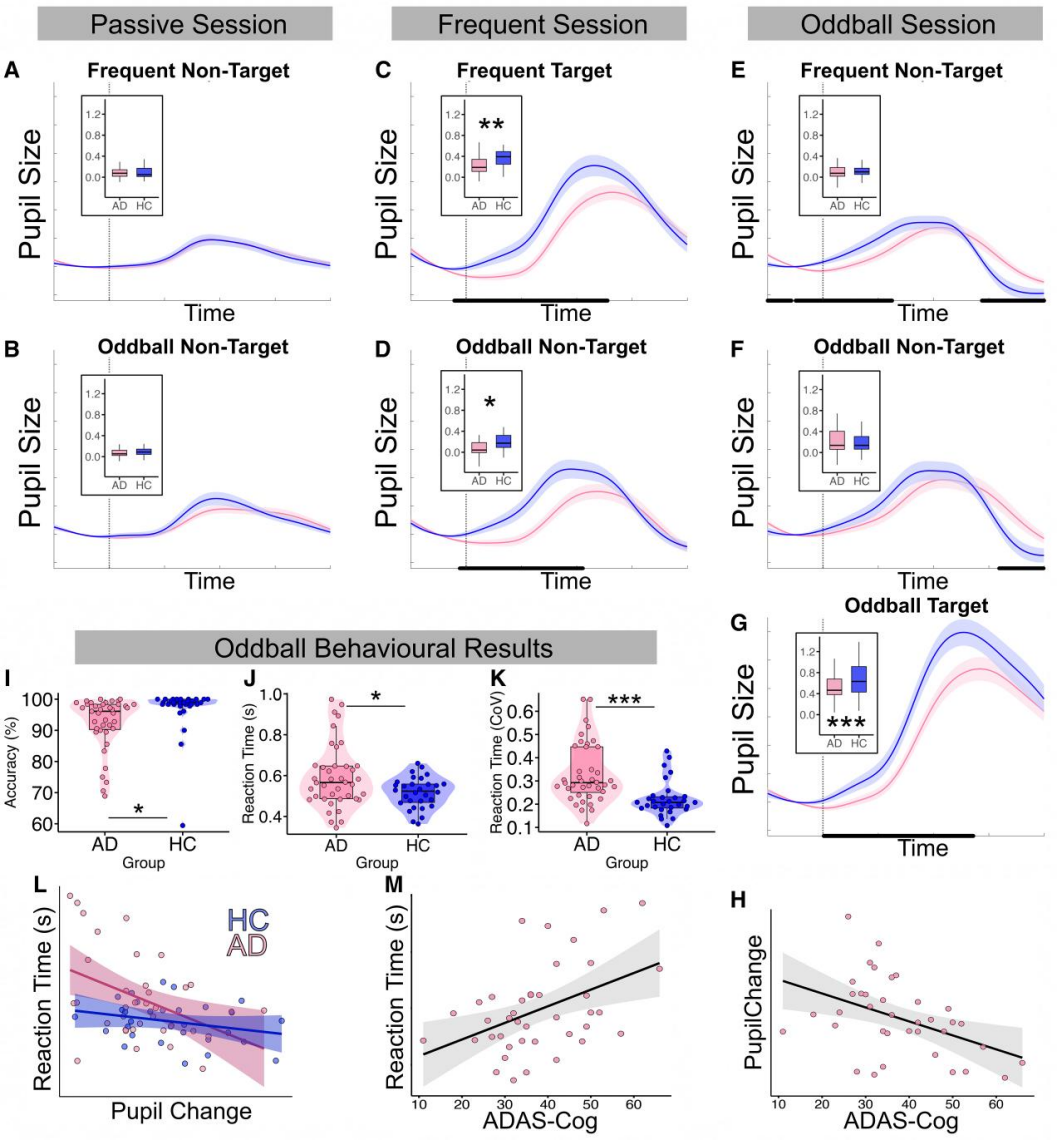
**Smaller, slower pupil dilation and slower and more variable reaction times, in Alzheimer’s disease.** (**A–G**) Group-average pupillary response curves for the seven trial types, showing pupil size from 0.5 s pre-stimulus to 2 s post-stimulus, normalized to a 0.5 s pre-stimulus baseline. Stimulus onset marked by vertical dashed line. Incorrect trials and those with incomplete data following blink interpolation excluded. Periods of significant difference between the lines (*t*-test) indicated by black bar. AD in pink and HC in blue. Insets show box plots comparing group-average pupil dilation responses (average baseline-corrected pupil size in 2 s post-stimulus period) with significant differences indicated where applicable. (**H**) Scatter plot showing relationship between ADAS-Cog total score and pupil dilation in response to oddball targets in AD group only (*b* = −0.01, *P* = 0.03). (**I**) Violin plot showing group difference in accuracy (percentage of trials correct from frequent and oddball sessions) (*b* = 4.37, *P* = 0.03). (**J/K**) Violin plots showing group differences in reaction times from all target trials (oddball and frequent) (reaction times: *b* = −0.07, *P* = 0.02; CoV: *b* = −0.10, *P* < 0.001). (**L**) Scatter plot showing relationship between pupil change (dilation to oddball target) and reaction time (all target trials), split by group. (**M**) Scatter plot showing relationship between ADAS-Cog total score and reaction time (all target trials) in AD group only (*b* = 0.01, *P* = 0.02). Significance displayed in **I–K** are results of linear regression including age and sex as covariates. Plotted line in scatter plots represents best fit through the raw data. Shaded area in line and scatter graphs = standard error of the mean. *P < 0.05; **P < 0.01; ***P < 0.001. AD, Alzheimer’s disease; HC, healthy control; ADAS-Cog, Alzheimer’s Disease Assessment Scale–Cognitive Subscale; CoV, coefficient of variation.

Pupillary responses, magnitude of response (‘pupil dilation response’) and time taken for the pupil size to reach a maximum (‘latency’) during oddball performance were used to quantify phasic arousal activity. All seven trial types induced a pupil dilation response relative to baseline in both groups (Fig. 6A–G). These responses varied depending on trial type. Investigation of pupil dilation responses showed significant main effects for trial type [F(6,384) = 97.68, *P* < 0.001, η² = 0.60] and a significant interaction between group and trial type [F(6,384) = 4.65, *P* < 0.001, η² = 0.07]. The main effect of group approached significance [F(1,64**)** = 3.61, *P* = 0.06, η² = 0.05], with controls having greater pupil dilation responses.

There were significant differences in pupil dilation response for four trial types compared to the reference condition (passive session frequent non-targets): the frequent session frequent targets (estimate = 0.29, SE = 0.04, *t* = 7.48, *P* < 0.001), oddball session oddball targets (estimate = 0.60, SE = 0.04, *t* = 15.54, *P* < 0.001), frequent session oddball non-targets (estimate = 0.13, SE = 0.04, *t* = 3.25, *P* = 0.001) and oddball session oddball non-targets (estimate = 0.11, SE = 0.04, *t* = 2.88, *P* = 0.004). When examining the interaction between group and trial type, significant contrasts were found for three trial types, including the two salient target trial types. Controls had significantly higher pupil dilation responses for the frequent session frequent targets (estimate = 0.14, SE = 0.05, *t* = 2.82, *P* = 0.005), frequent session oddball non-targets (estimate = 0.11, SE = 0.05, *t* = 2.29, *P* = 0.02) and the oddball session oddball targets (estimate = 0.19, SE = 0.05, *t* = 3.92, *P* < 0.001) (Fig. 6A–G). After false discovery rate correction, the *P*-values were 0.02, 0.05 and <0.001, respectively. Figure 6 shows how the magnitude of the pupil dilation response was greater in the control group for these three trial types in the first 1.5 s post-stimulus in particular.

We replicated this analysis using latency, as a measure of the speed of phasic response. There were significant effects of trial type [F(6,384) = 75.58, *P* < 0.001, η² = 0.54] and group [F(1,64) = 4.23, *P* = 0.04, η² = 0.06], with overall shorter latencies for the control group. The interaction between group and trial type was not significant (*P* > 0.05). The model’s fixed effects revealed significant differences in latency for three types compared to the reference condition. In AD, there was a longer latency for frequent session frequent targets (estimate = 88.66, SE = 18.18, *t* = 4.88, *P* < 0.001) and oddball session oddball targets (estimate = 173.67, SE = 18.18, *t* = 9.55, *P* < 0.001), but a shorter latency for oddball session frequent non-targets (estimate = −50.54, SE = 18.18, *t* = −2.78, *P* = 0.006) (Fig. 6A–G).

We used pupil dilation response to the targets in the oddball session as a measure of an individual’s phasic activity as this stimulus type was designed to induce the greatest arousal response (infrequent and salient). Reaction time across the whole task predicted pupil dilation response to oddball session target trials when accounting for group (*b* = −0.67, SE = 0.33, *t* = −2.02, *P* < 0.05), with no significant group interaction (*P* > 0.05). In the AD group, worse ADAS-Cog score predicted reduced pupil dilation response to oddball session target trials (*b* = −0.01, SE < 0.01, *t* = −2.30, *P* = 0.03) (Fig. 6H).

Unpaired *t*-tests did not show any significant difference between AD participants on acetylcholinesterase inhibitors (*n* = 28/40) and those not, for any of LC contrast, peak alpha frequency, ADAS-Cog, oddball target pupil dilations or latency or any behavioural measures from the oddball task (all *P* > 0.05) (see Supplementary Results for full statistics).

## Discussion

Our results demonstrate a range of structural and functional arousal system abnormalities in patients with AD. We thoroughly investigated this using simultaneous pupillometry-EEG and an MRI sequence used to show contrast in the LC. Peak alpha frequency, a measure of tonic arousal, was reduced during rest and was associated with abnormal LC contrast. Measures of phasic arousal during an oddball task were also affected by AD and related to cognitive impairment. The results show that fluctuations in arousal state can be effectively measured using pupillometry^23^ and provide evidence for strong relationships between pupil size and EEG power in both groups, but that the directionality of the relationship is affected by cortical slowing in AD. Overall, we show that pupillometry represents a valuable tool with potential clinical utility.

Animal work has shown a clear relationship between pupil size and arousal nuclei activity.^22,23,25^ Pupil-linked arousal was abnormal in AD, and this effect could not be explained by low-level ocular dysfunction. Patients did not have abnormalities in basic pupil dynamics at rest, and redilation velocity following light-induced constriction is equal or increased in AD, implying that the disease does not cause ophthalmic inhibition of dilation mechanisms.^62^

We saw very strong correlations between spontaneous pupil dynamics and EEG rhythms. This relationship has previously been reported in healthy adults, and our results show that it exists in a distinct form in AD. Specifically, the relationship between pupil size and multiple EEG measures of arousal was seen in both groups: suppressed low-frequency (alpha and below) power^63,64^ and a reduced aperiodic exponent,^44^ plus in AD only, an increased peak alpha frequency.^21^ All three reflect a shift to higher-frequency power as arousal increases and highlight the value of pupillometry to probe the effect of AD on cortical dynamics.^22,30,35,41^ Interestingly, the direction of the pupil/EEG power relationship is frequency, lag and group dependent.^41^ Cortical slowing, an established consequence of AD, affected this relationship.^37,39^ At a −2 s lag, the relationship between pupil size and low-frequency power is positive in controls but negative in AD (Fig. 3E), with the direction related to an individuals’ peak alpha frequency. The spontaneous rise and fall seen in pupil-linked arousal and alpha power during rest are temporally aligned, but cortical slowing and sluggish pupil responses in AD affect this alignment (Fig. 3E; Supplementary Fig. 2).

We did not find group-level alterations in the aperiodic EEG component.^65^ However, in addition to the periodic band-power component of the EEG, we found very strong relationships between pupil size and the aperiodic component, corroborating previous MEG observations^41^ and extending these findings to AD (Fig. 4). This shows the influence of the arousal system on the aperiodic component, and there may be AD-related abnormalities we have not identified. Quantifying arousal-related fluctuations in the aperiodic component during a task and its relationship with cognitive performance in AD could be a focus for future work.^42,45^

Having investigated tonic arousal fluctuations during rest, we also used an oddball task to study phasic arousal responses by measuring the speed and magnitude of pupil responses to arousing stimuli. We replicated work showing extremely strong, negative correlations between pre-stimulus pupil size and dilation response to stimuli, in both groups (Fig. 5A and B). Importantly, this has been shown to reflect the balance between tonic and phasic activity, rather than a function of baseline pupil size *per se*.^66^ As in resting-state, greater pupil-linked tonic arousal was associated with suppressed alpha power which itself had a positive effect on performance in both groups (Fig. 5B).^36,40^ More salient stimuli (targets) induced greater dilations, but more so in controls than AD (Fig. 6A–G). One explanation is that patients with AD fail to mount a healthy phasic arousal response when needed. An alternative explanation might be that the AD group’s tonic arousal was higher, and therefore, phasic activity was suppressed.^66^ Unfortunately, as the pupil time courses were *Z*-scored to account for between-subject differences in absolute pupil size not reflective of arousal state, comparison between subjects of baseline pupil size was not possible. Regardless, the magnitude of phasic response was related to overall cognition in the AD group (Fig. 6H), providing further evidence that this reduced responsiveness to important external stimuli has relevance for patients’ cognitive symptoms. The speed of the pupil response to task stimuli was also reduced in AD which reflects responses to spontaneous fluctuations in the alpha time course during rest (Supplementary Fig. 2E and F). This reinforces the evidence of an attenuated arousal system in AD that cannot respond as briskly and dynamically when required, to both internally generated fluctuations and external stimuli.

There may be an evolutionary explanation for the tight coupling of pupillary responses and brain activity. The LC is present in all mammals and as such is evolutionarily primitive. As the neocortex evolved, the existing neuromodulatory components of the autonomic nervous system may have been co-opted for the purpose of widespread tuning and higher-order cognition.^67^ Arousal-modulating neurotransmitters including noradrenaline and acetylcholine broadcast to a range of structures have a relatively weak effect on synaptic transmission and modulate brain-states over longer timescales, making them suited to diverse neuromodulatory effects.^68-70^ It is possible that LC projections have evolved to have complementary effects on pupillary responses and brain network dynamics, i.e. contributing to the flight-or-fight response. The very tight relationship between pupillary response and arousal-related EEG fluctuations would be in keeping with a common evolutionary development. Pupil dilation increases light reaching the retina, which can increase the ability to sense salient visual information in some situations, although is associated with a reduction in visual acuity.^71^ Hence, changes in pupil size could merely reflect a read-out of arousal level, rather than representing functional changes for the purpose of improving behavioural performance.

This study has limitations to consider. One potential confound is that some of the AD group were on acetylcholinesterase inhibitors, potentially affecting pupillometry and cortical measures.^2,22^ However, their use in a proportion of patients did not seem to affect results. Also, it is possible that the use of acetylcholinergic drugs in some of the patients actually reduced the differences seen between AD and controls. Furthermore, our study better reflects the real-world reality of AD care and future therapeutic modulation of the noradrenergic system will likely be in conjunction with existing treatments that have their own neuromodulatory effects. Secondly, we may have benefitted from longer inter-stimulus intervals in the oddball task to establish baseline measures of arousal state that were not reliably measurable in the current design, such as peak alpha frequency and aperiodic exponent. This represents a trade-off against task length and risk of reduced tolerability. Thirdly, it is unlikely that resting-state and a relatively simple task would elicit the full spectrum of arousal. However, our results show that even in our paradigms, evidence of dysfunction is demonstrable in AD and relevant to subtle fluctuations in arousal that occur in everyday life.

In summary, we show structural and functional deficits in the arousal system of patients with AD. Arousal fluctuations at rest are abnormal in AD as measured by combined pupillometry and EEG. During task, salient stimuli that require a behavioural response are accompanied by a phasic increase in arousal, demonstrated by pupil dilation to oddball stimuli. This response is slower and of smaller magnitude in AD patients. These reductions are correlated with slower behavioural responses and abnormalities in cognition. Cortical slowing (reduced peak alpha frequency) is seen in AD, and this is modulated by arousal level and relates to overall cognition. Pupil-linked arousal responses and EEG fluctuations are normally tightly coupled, and cortical slowing produced by AD influences this coupling. The tools used here have potential in not just understanding the nature of arousal system dysfunction in AD at the group level, but also as biomarkers at the individual level as part of clinical care that may utilize arousal-modulating treatments.^19,72^

## Supplementary Material

fcaf236_Supplementary_Data

## Data Availability

All data associated with this study are present in the paper or the Supplementary Materials. Raw, unprocessed data are available from the corresponding author upon reasonable request and following the completion of a material transfer agreement (MTA) with Imperial College, London.

## Appendix I

**Authors in the CR&T Group of UKDRI**

Professor David Sharp

Danielle Wilson

Sarah Daniels

Professor Ramin Nilforooshan

Matthew Harrison

Dr Shlomi Haar

Dr Mara Golemme

Professor Payam Barnaghi

Professor Paul Freemont

Professor Ravi Vaidyanathan

Professor Tim Constandinou

Dr Gregory Scott

Professor Derk-Jan Dijk

Dr Pete Lally

Professor Paresh Malhotra

Dame Professor Louise Robinson

Professor Adam Hampshire

Michael David

Martina Del Giovane

Neil Graham

Magdalena Kolanko

Helen Lai

Lucia M Li

Mark Crook Rumsey

Emma Jane Mallas

Alina-Irina Serban

Eyal Soreq

Abidemi Otaiku

Megan Parkinson

Thomas Parker

Success Fabusoro

Emily Beal

Alan Bannon

Danilo Mandic

Ziwei Chen

Charalambos Hadjipanayi

Ghena Hammour

Bryan Hsieh

Amir Nassibi

Adrien Rapeaux

Ian Williams

Maowen Yin

Niro Yogendra

Maria Lima

Ting Su

Melanie Jouaiti

Maitreyee Wairagkar

Carlos Sebastian Castillo

Panipat Wattansiri

Thomas Martineau

Mayue Shi

Tianbo Xu

Bo Xiao

Alejandro Valdunciel

Reneira Seeamber

Annika Guez

Zehao Liu

Saksham Dhawan

Nan Fletcher-Lloyd

Samaneh Kouchaki

Alexander Capstick

Chloe Walsh

Louise Rigny

Ruxandra Mihai

Marirena Bafaloukou

Jin Cui

Ann-Kathrin Schalkamp

Yu Chen

Tianyu Cui

Nivedita Bijlani

Michael Crone

Kirsten Jensen

Martin Tran

Thomas Adam

Raphaella Jackson

Alexander Webb

Anne C Skeldon

Kevin Wells

Ullrich Bartsch

Ciro Della Monica

Kiran K G Ravindran

Damion Lambert

Sara Mohammadi Mahvash

Thalia Rodriguez Garcia

Victoria L Revell

Giuseppe Atzori

Lucinda Grainger

Hana Hassanin

James Woolley

Iris Wood-Campar

Janetta Rexha

Sarmad Al Gawwam

Subati Abulikemu

Julian Jeyasingh Jacob

Nathan Steadman

Federico Nardi

Cosima Graef

Alena Kutuzova

Assaf Touboul

Nicolas Calvo Peiro

Jenna Yun

Gaia Frigerio

Adela Desowska

Anastasia Gailly de Taurines

Ruxandra Mihai

Nina Moutonnet

Matthew Harrison

Sophie Horrocks

Brian Quan

Mark Woodbridge

Anna Joffe

Amer Marzuki

Ramsheed Abdul Rahim

Professor Ramin Nilforooshan

Jessica True

Olga Balazikova

Nicole Whitethread

Matthew Purnell

Vaiva Zarombaite

Lucy Copps

Olivia Knight

Gaganpreet Bangar

Sumit Dey

Chelsea Mukonda

Jessica Hine

Luke Mallon

Saijal Jhala

Oliver Sargentoni

Amy Alves

Mahan Heydari

Dr David Wingfield

Claire Norman

Anesha Patel

Ruby Lyall

Sanara Raza

Naomi Hassim

Pippa Kirby

John Patterson

Mike Law

Andy Kenny
