# Supplementary material for: Pupil-linked arousal, cortical activity, and cognition in Alzheimer’s disease
Source: Brain Commun. 2025 Jul 22;7(4):fcaf236. doi: 10.1093/braincomms/fcaf236 (PMC12280328; doi:10.1093/braincomms/fcaf236)
Supplement: fcaf236_Supplementary_Data [file fcaf236_supplementary_data.docx]

**Supplementary Materials**

**Supplementary Methods**

# **MRI acquisition**

Participants underwent a T1-weighted three-dimensional magnetisation-prepared rapid acquisition gradient-echo (3D-MPRAGE) sequence. The parameters were: flip angle=9º, echo time=2.98ms, repetition time=2300ms, inversion time=900ms, bandwidth=240Hz/pixel, acquisition matrix=256x240x160, voxel size=1.0x1.0x1.0mm, GRAPPA acceleration factor=2.

LC contrast was quantified using the following MRI sequence (3D T2*-weighted multi-echo gradient-echo with magnetisation transfer preparation pulse) which was aligned perpendicularly to the plane of the participant’s brainstem based on the sagittal reconstruction from the MPRAGE sequence and used the following parameters: flip angle=20º, echo times=7.5,15.0,22.5ms, repetition time=62ms, acquisition matrix=384x384x48, voxel size=0.67x0.67x1.34mm, bandwidth=230Hz/pixel, GRAPPA acceleration factor=2, slice partial Fourier factor=6/8. The first echo time was analysed.

**Pupillometry-EEG assessment set-up**

Participants underwent simultaneous pupillometry and EEG during 5mins of eyes-open resting-state and then during an oddball task, conducted in a windowless room, shielded with a Faraday cage. Paradigms were run using MATLAB R2021a (MathWorks). High-density EEG was recorded continuously using a 256-channel hydrocel geodesic sensor net (Electrical Geodesics, Inc. [EGI]), following sizing and soaking of the cap in potassium chloride solution as per manufacturer instructions. The EEG was recorded on the EGI Net Station Acquisition 5.4.3 offline at a sampling frequency of 1000Hz and was amplified using the Net Amps 400 system. Electrode impedance in all channels (except those at the nape of the neck) was verified to be <100kΩ, according to EGI guidelines, before starting the recording. The pupil diameter (from here on referred to as size) was measured using an EyeLink 1000 Plus Desktop Mount eye-tracker equipped with the High Speed 35mm lens (focal length of 2.4) (SR Research Ltd., Canada) from the right eye. Participants were seated 100cm from a 24” LCD screen with their chin on a chinrest, and the camera was placed midway between them and the screen. A 5-point calibration procedure was conducted prior to the resting-state and task to ensure accurate gaze-position tracking.

**Oddball paradigm**

The oddball task involved participants listening to a sequence of auditory stimuli, consisting of spoken letters B, K, and M played through the monitor speaker. The task paradigm was divided into three ‘sessions’. Letters were used rather than tones as higher-pitched tones can be less well heard, especially by older people,^1^ and B, K, and M were chosen as they have diverse auditory profiles and are therefore easy to distinguish from one-another. The stimuli were generated using Audacity^2^ with a consistent volume and 300ms duration. The screen and room brightness were consistent throughout and across participants. The task paradigm is represented in Figure 1. One letter was played for 60% of trials (‘frequent’) and the other two letters were played for 20% of trials each (‘oddballs’). Trials were separated by an inter-stimulus interval randomly jittered between 2.3-2.7s. The letters were played in a pseudorandom order with the stipulation that there was never more than seven consecutive frequent and three consecutive oddball trials. The three task sessions were separated by a short break of up to 1min. Each session contained two blocks of 45 trials separated by a 10s break. The first two trials were discarded for analysis, producing a total of 268 trials. Throughout the task, participants were instructed to stare at the central fixation cross. The first session was the ‘passive session’, in which participants simply listened to the (‘non-target’) stimuli. For the second and third sessions, participants were asked to respond to an assigned letter (‘targets’) by pressing a button, and not others (‘non-targets’). For one of these two sessions, the target stimuli were frequently occurring (‘frequent session’), and for the other, they were one of the two oddball stimuli (‘oddball session’). In the oddball session, one letter represented a non-target oddball. The order of the frequent and oddball sessions, and the use of each of the letters as oddballs/targets was randomly assigned across participants using a Latin Square. This was done to negate potential order effects or effects of the letters themselves. Written instructions regarding required response to stimuli were displayed on the screen at the beginning of each block, and verbal reminders were given during the run, up to twice per session, if participants were making consistent errors.

**EEG data pre-processing**

For the resting-state, data were segmented into 20s segments, whereas for the oddball task, the data were segmented into 2.5s segments beginning 0.5s before the stimulus onset. Otherwise, the pre-processing was as follows for both datasets: 55 of the 256 channels, located on the back of the neck and on the face, were excluded as channels of no interest. Data were downsampled to 250Hz then bandpass filtered at 1-100Hz. Electrical line noise at 50Hz was removed using the CleanLine multitaper approach.^3^ Of the remaining 201 channels, those with poor signal quality were rejected using the default EEGLAB Clean Rawdata function with optimised criteria. Data from rejected channels were re-interpolated. Segments were then rejected based on probability criteria, jointly using an amplitude-based artifact threshold (+/-200mV) and segment similarity criteria (using EEGLAB’s ‘jointprob’ function) which considers how likely a segment is to be artefact-laden based on activity of other segments for that channel. Finally, bad channels were re-interpolated and data were re-referenced to average.

**Pupil data pre-processing**

Blinks of ≤0.5s duration were interpolated using a piecewise cubic Hermite interpolating polynomial function. Data were bandpass filtered between 0.05-2Hz to reduce high-frequency noise and slow drift that may be the result of participant movement, whilst still including the frequency range of arousal-linked pupillary phenomena.^4,5^ Data were then ﻿z-scored as this has been shown to elicit a uniformed inter-subject variability between task conditions, while maximising differences between task conditions.^6^

Pupil Size (a.u., z-scored)

Pupil Size (a.u., z-scored)


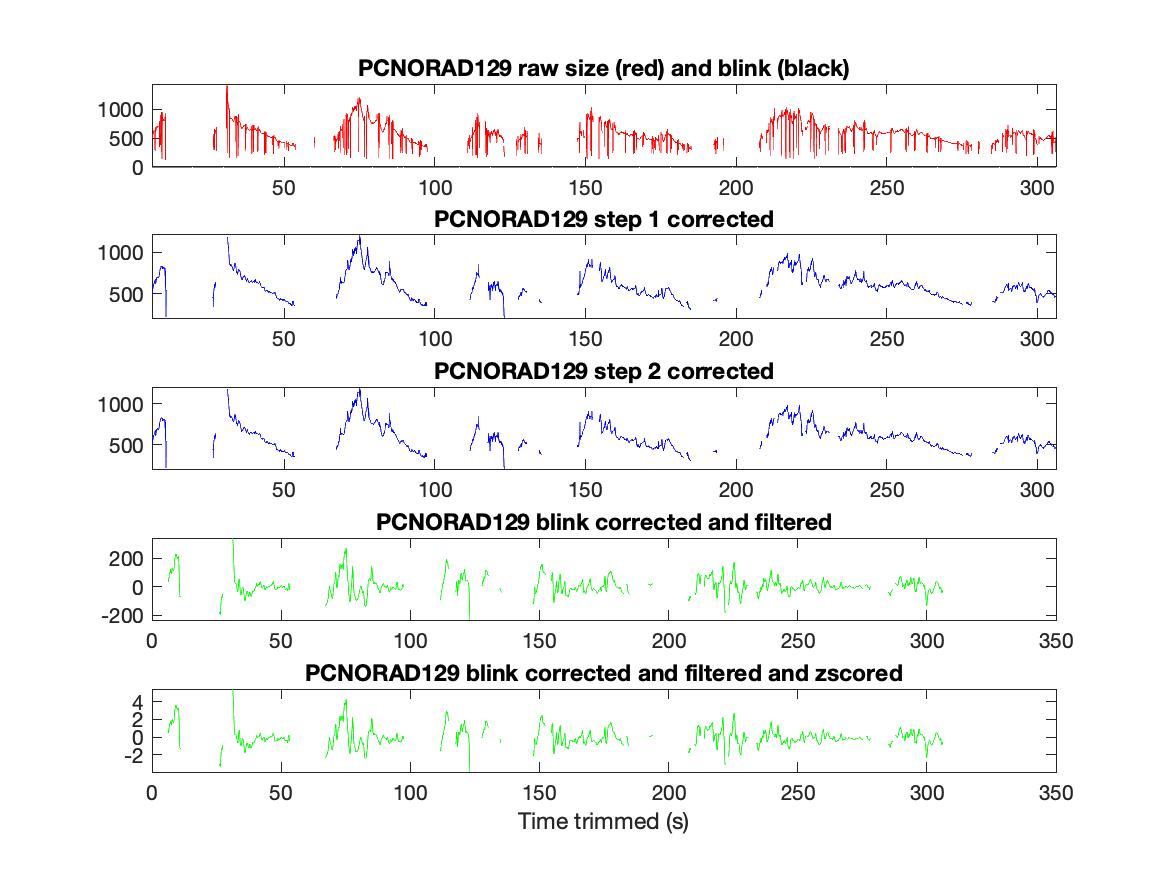

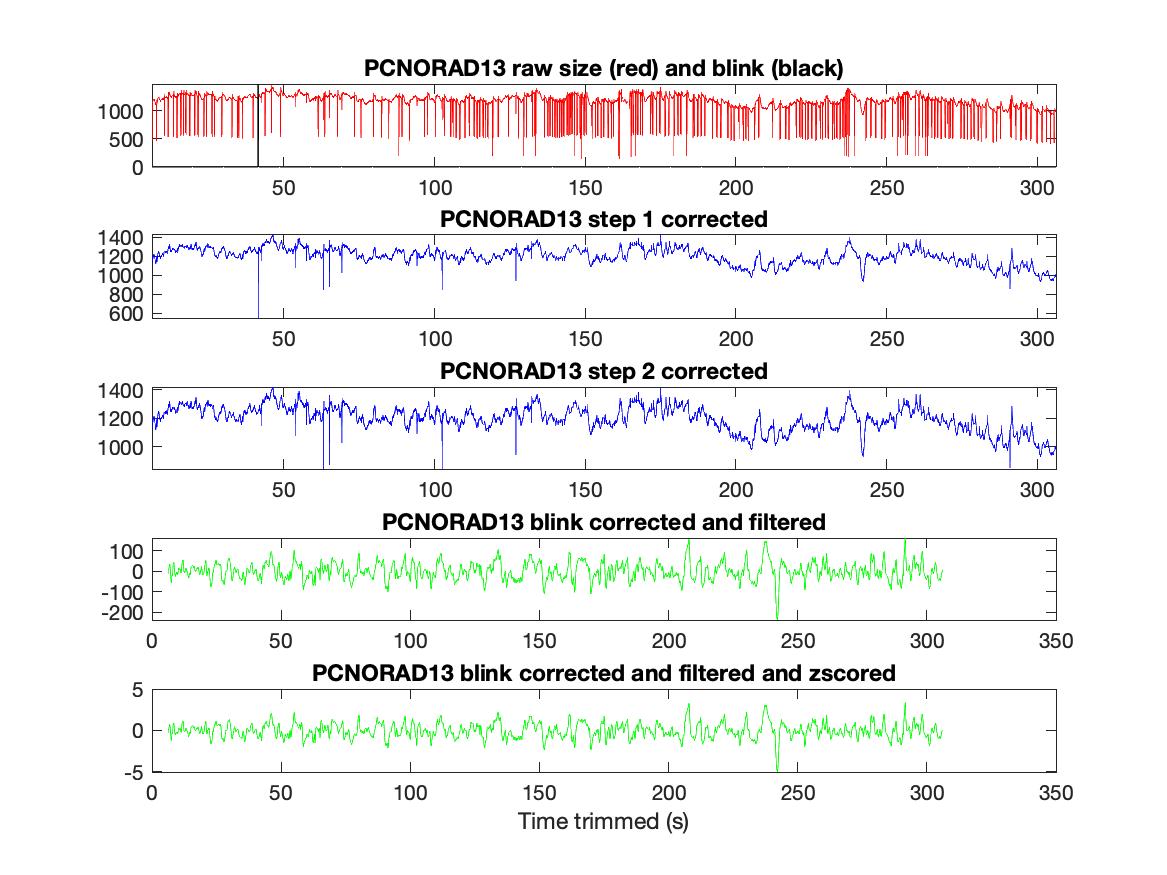


**A)**

**B)**

***Supplementary Figure 1. Examples of resting-state pupil data. A)*** *An example of good data post processing.* ***B)*** *shows an example of a rejected participant due to poor data.*

**Locus coeruleus contrast quantification**

The LC-sensitive MR images were biased corrected using N4BiasFieldCorrection by ﻿Advanced Normalization Tools v2.1 (ANTs). The location of the LC is identifiable in the pons as two hyperintense areas around the base of the fourth ventricle.^7^ Using an axial plane, a middle slice was chosen, located ﻿7mm below the inferior boundary of the inferior colliculus.

Extending the previously reported method we also examined two slices above and below the middle LC slice, giving a total of five slices.^8^ The method by Clewett et al. that we extended, was applied to data with 2.5mm slice thickness and 3.5mm slice gap, whereas in our data we have a 1.34mm slice thickness with no gap between slices. This allowed for us to sample the LC (which is 12-17mm long)^9^ across multiple slices, providing a more reliable value per individual and improving the signal-to-noise ratio. Also, given the known rostral-caudal gradient in AD related changes in the LC^10^, it was pertinent to sample the length of the nucleus. Using ﻿FMRIB’s Software Library (FSL), a 5 voxel, cross-shaped region of interest was chosen, centred on the highest intensity voxel within the putative LC region, on the right and left. In instances where the most intense voxel was adjacent to a voxel in the fourth ventricle (defined as a voxel with >50 lower intensity than the central LC voxel), the centre of the region of interest was moved one voxel in the medial or rostral direction (to whichever was more intense). The average signal intensities were calculated from the 5 LC voxels on each side. A square shaped 7x7 voxel reference region was produced, where the centre of the square was 8 voxels in the rostral direction from the average of the centre of the two LC regions and equidistant between them.^8^ The contrast ratio in the LC was measured as a ratio compared to the reference regions in the nearby pons for each slice separately calculated using the following equation: (mean of left and right LC contrast – mean reference contrast)/standard deviation of reference contrast.^7^ Then, this contrast-to-noise ratio was averaged for the five slices, yielding a single overall contrast measure.

**Peak alpha frequency analysis**

Peak alpha frequency was calculated using the ‘restingIAF’ function,^11^ specifically the centre of gravity metric. Centre of gravity is the weighted sum of spectral estimates divided by the total alpha power.^11^ By measuring the central tendency of the alpha power, the centre of gravity is therefore considered more representative of the average alpha activity.^12^ This is particularly useful if there are multiple peaks in the alpha range. This function uses pwelch, normalisation and Savitzky–Golay filter smoothing (11 bins, polynomial degree of five), and the global peak alpha frequency was estimated using a minimum of three valid channels (i.e. with reliable peaks), as per recommendations. We extended the frequency search window from the recommended 7-13Hz to 5-14Hz.^11^ This function provided a single peak alpha frequency per participant across the whole resting-state, and at the level of pupil bin for each participant, for the separate analysis of peak alpha frequency as a function of pupil size.

**Aperiodic EEG component analysis**

To separate out the aperiodic component of the power spectra, we used the FieldTrip wrapper for the MATLAB implementation of ‘FOOOF’ in the Brainstorm toolbox.^13^ This function decomposes the power spectrum into the aperiodic component, calculated using the Lorentzian function with an offset and an exponent, and a periodic component defined by identifying Gaussian peaks reflective of oscillations. We used the default settings as defined in the process_fooof MATLAB function, except the frequency range used was 3-48Hz. This is an extension of the 3-40Hz range used by others when comparing aperiodic power to pupil size,^5^ on the basis of evidence that the exponent of the spectrum in the gamma range in particular relates to E/I ratio.^14^

**Statistical Analysis**

All analysis was conducted using RStudio Version 2024.04.2+764 (Posit, PBC) and MATLAB R2021a (MathWorks). Group differences in demographics and pupil data metrics (skewness, kurtosis, number of peaks/troughs) were compared using t-tests, except for sex for which chi-square was used.

For analysis of the relationship between EEG band power and pupil decile, linear and quadratic models were fitted and tested for significance at P<0.05. Bayesian information criterion (BIC) was used to test whether a simpler model was preferred at risk of overfitting in the case of the quadratic model. If both models were significant, the model with the lower BIC was plotted. A linear mixed-effects model was employed to test for the effect of group and pupil deciles on the aperiodic exponent, as well as their interaction. The model included a random intercept for individual participants to account for repeated measures within each participant. The model was fitted using the ‘lmer’ function from the lme4 package in RStudio.

For quintile-based analysis of pupil, EEG, and reaction time measures in the oddball task, linear mixed-effects models were used to test for linear and U/inverted U-shaped relationships by adding a quadratic term into the model.^15^ Alpha power and behavioural responses were rank normalised due to skewed distributions. Significant linear and quadratic models (P<0.05) were plotted and compared using Bayesian information criterion. For analysis of pupil dilation responses, only correct trials were included. For these, we also employed a linear mixed-effects model, with the pupil metric (dilation response/time to maximum) as the dependent variable and group, trial type, and their interaction as fixed effects. A random intercept for participant was included to account for individual variability among participants. Subsequently, an ANOVA was performed to assess the significance of the fixed effects. To quantify the magnitude of these effects, we calculated partial eta squared values. Post-hoc analyses were conducted using the ‘emmeans’ function in R to perform pairwise comparisons of the trial types. Further pairwise comparisons of the group levels across different trial types were used to explore interaction effects.

**Supplementary Results**

| **Pupil Decile Against EEG Band Power** | | | | | |
| --- | --- | --- | --- | --- | --- |
| **Group** | **EEG band** | **Shift (s)** | **Model** | **Adj R^2^** | **P-value** |
| HC | Delta | 0 | L | -0.13 | 0.99 |
|  |  |  | Q | -0.04 | 0.48 |
|  |  | 1 | L | **0.90** | **<0.001*** |
|  |  |  | Q | **0.89** | **<0.001** |
|  |  | 2 | L | **0.50** | **0.01** |
|  |  |  | Q | **0.69** | **0.007*** |
|  | Alpha | 0 | L | -0.10 | 0.67 |
|  |  |  | Q | **0.80** | **0.001*** |
|  |  | 1 | L | **0.89** | **<0.001*** |
|  |  |  | Q | **0.90** | **<0.001** |
|  |  | 2 | L | **0.55** | **0.008*** |
|  |  |  | Q | **0.53** | **0.03** |
|  | Beta | 0 | L | **0.70** | **0.002** |
|  |  |  | Q | **0.74** | **0.004*** |
|  |  | 1 | L | -0.04 | 0.44 |
|  |  |  | Q | 0.29 | 0.12 |
|  |  | 2 | L | -0.07 | 0.53 |
|  |  |  | Q | 0.19 | 0.19 |
| AD | Delta | 0 | L | -0.05 | 0.47 |
|  |  |  | Q | -0.12 | 0.61 |
|  |  | 1 | L | **0.84** | **<0.001** |
|  |  |  | Q | **0.95** | **<0.001*** |
|  |  | 2 | L | **0.54** | **0.009** |
|  |  |  | Q | **0.85** | **<0.001*** |
|  | Alpha | 0 | L | **0.41** | **0.03*** |
|  |  |  | Q | 0.38 | 0.08 |
|  |  | 1 | L | **0.83** | **<0.001** |
|  |  |  | Q | **0.87** | **<0.001*** |
|  |  | 2 | L | **0.56** | **0.008*** |
|  |  |  | Q | **0.53** | **0.03** |
|  | Beta | 0 | L | **0.83** | **<0.001** |
|  |  |  | Q | **0.91** | **<0.001*** |
|  |  | 1 | L | -0.12 | 0.88 |
|  |  |  | Q | -0.28 | 0.97 |
|  |  | 2 | L | 0.12 | 0.18 |
|  |  |  | Q | 0.09 | 0.29 |

***Supplementary Table 1. Results of linear and quadratic models of pupil decile against EEG power, as shown in Figure 3E.*** *Results of linear models (L) and models with quadratic component (Q) fitted to group level data of pupil decile against relative EEG power for the three bands of interest. Models fitted to unshifted pupil data (0s) and pupil data shifted forward by 1s and 2s. Significant models (P<0.05) highlighted in bold. Where relationship is significant, preferred model by lower Bayesian information criterion highlighted with *.*

| **Pupil Decile Against Peak Alpha Frequency** | | | | |
| --- | --- | --- | --- | --- |
| **Group** | **Shift (s)** | **Model** | **Adj R^2^** | **P-value** |
| HC | 0 | L | 0.01 | 0.32 |
|  |  | Q | 0.19 | 0.20 |
|  | 1 | L | 0.06 | 0.24 |
|  |  | Q | 0.02 | 0.38 |
|  | 2 | L | -0.11 | 0.73 |
|  |  | Q | -0.23 | 0.85 |
| AD | 0 | L | 0.08 | 0.27 |
|  |  | Q | 0.19 | 0.20 |
|  | 1 | L | **0.70** | **0.002*** |
|  |  | Q | **0.69** | **0.007** |
|  | 2 | L | 0.07 | 0.23 |
|  |  | Q | -0.06 | 0.51 |

***Supplementary Table 2. Results of linear and quadratic models of pupil decile against peak alpha frequency.*** *Results of linear models (L) and models with quadratic component (Q) fitted to group level data of pupil decile against peak alpha frequency. Models fitted to unshifted pupil data (0s) and pupil data shifted forward by 1s and 2s. Significant models (P<0.05) highlighted in bold. Where relationship is significant, preferred model by lower Bayesian information criterion highlighted with *. Results from 1s shift plotted in Figure 3D.*


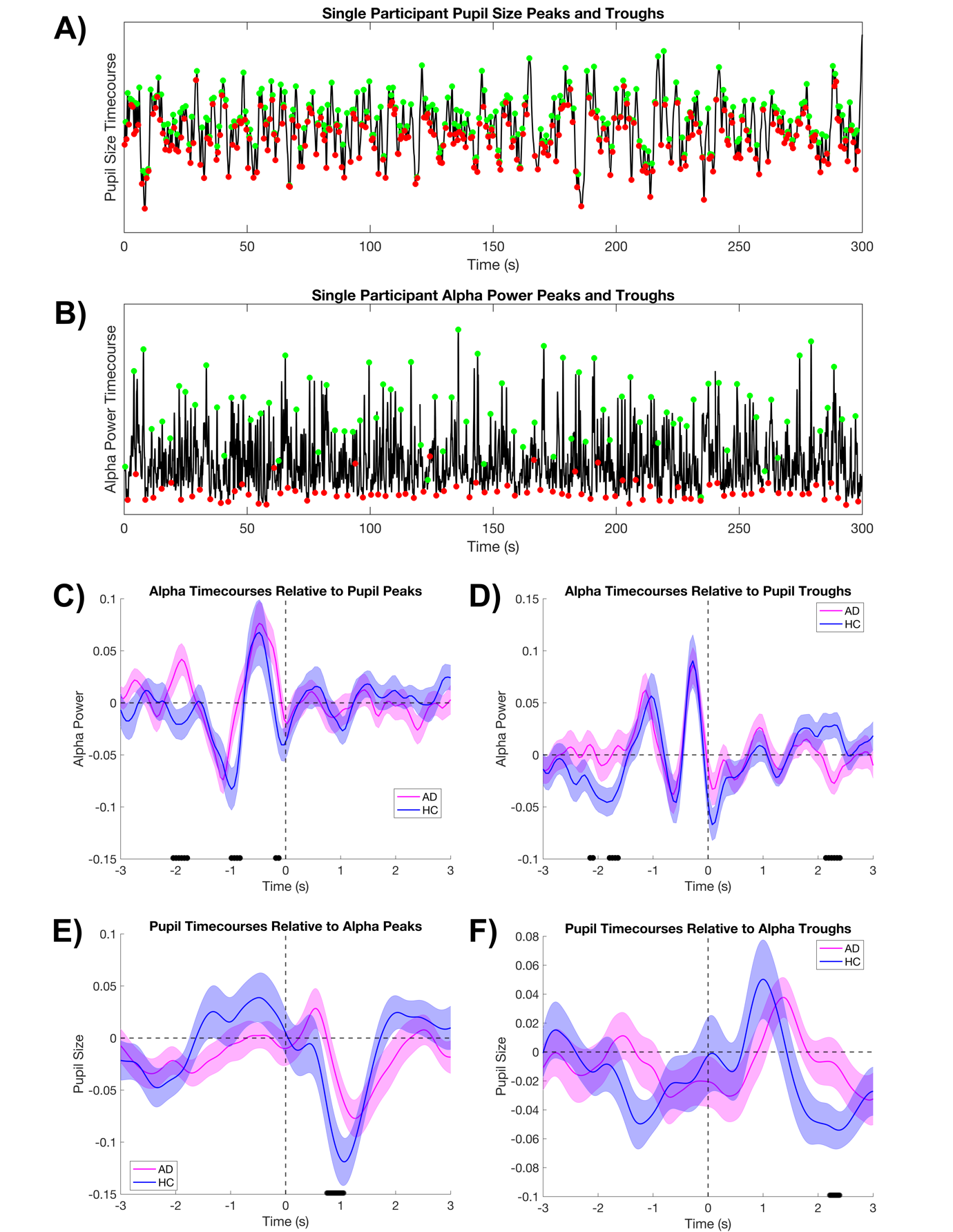


***Supplementary Figure 2. Pupil and alpha timecourses in relation to spontaneous peaks and troughs.*** ***A)*** *Example 5-mins resting-state pupil timecourse from one participant with spontaneous peaks (green) and troughs (magenta) identified.* ***B)*** *as in* ***A)*** *but for alpha power timecourse.* ***C)*** *Alpha power timecourses relative to spontaneous pupil peaks (vertical dashed line), averaged across all peaks, for all participants, separated by group.* ***D)*** *as* ***C)*** *but for pupil troughs.* ***E)*** *Pupil size timecourses relative to spontaneous alpha power peaks (vertical dashed line), averaged across all peaks, for all participants, separated by group.* ***F)*** *as* ***E)*** *but for alpha troughs. Periods of significant difference (P<0.05) between the lines (t-test) indicated by black bar. AD in pink, HC in blue. Shaded area = standard error of the mean. AD = Alzheimer’s disease, HC = healthy control.*


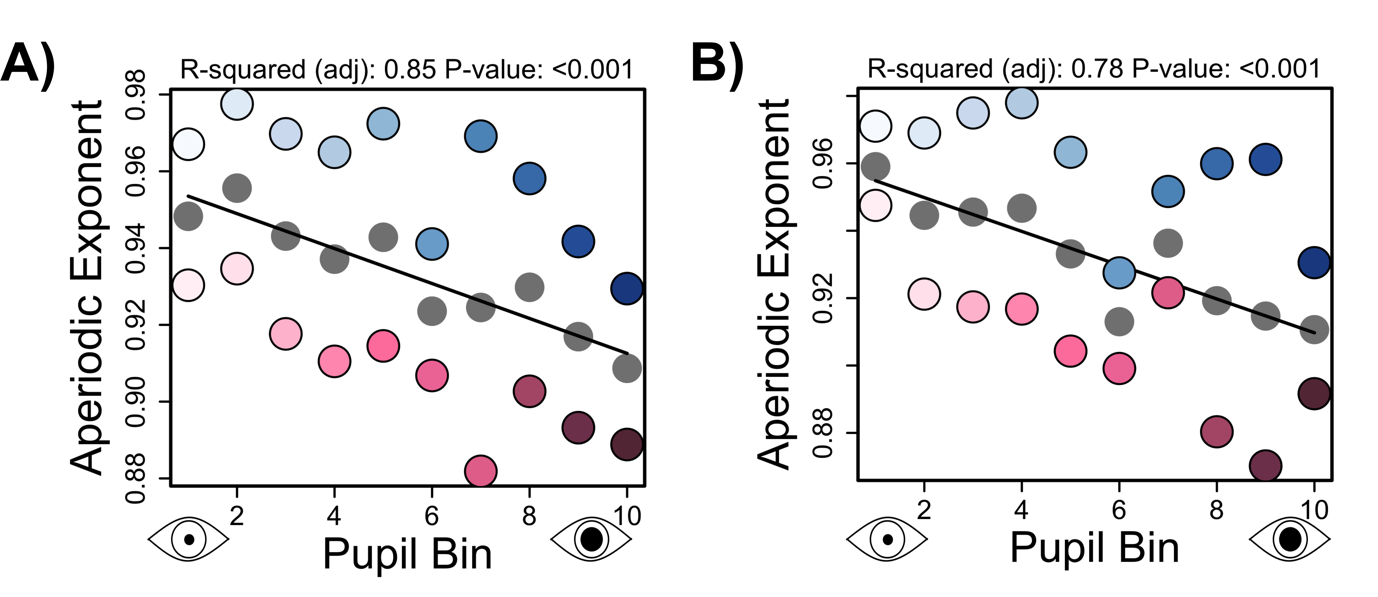


***Supplementary Figure 3. Pupil size relates to aperiodic EEG power.*** ***A)*** *Exponent of aperiodic power as a function of pupil size at 0s lag, shown for all participants (grey) and split by group (HC in blue, AD in pink).* ***B)*** *as in* ***A)*** *but at -2s lag. Best fit line, adjusted R-squared and P-values refer to grey scatter points. AD = Alzheimer’s disease, HC = healthy control.*

**Comparison of AD patients on and off acetylcholinesterase inhibitors**

On t-testing, was no significant difference between those on acetylcholinesterase inhibitors for any of the following metrics: ADAS-Cog [t(17.41)=0.80, P=0.44]; LC contrast [t(15.05)=-0.20, P=0.85]; peak alpha frequency [t(16.52)=-2.05, P=0.06]; oddball target pupil dilation response [t(21.32)=-0.07, P=0.95] or latency [t(25.74)=-0.61, P=0.55]; oddball task accuracy [t(36.76)=1.56, P=0.13], reaction time [t(31.83)=-0.17, P=0.86], or CoV of reaction time [t(25.81)=-1.02, P=0.32].

**Supplementary references**

1. Bunch CC. AGE VARIATIONS IN AUDITORY ACUITY. Arch Otolaryngol. 1929;9(6):625–36.

2. Audacity(R). Software is copyright (c) Audacity Team. [Web site: http://audacity.sourceforge.net/. It is free software distributed under the terms of the GNU General Public License.] The name Audacity(R) is a registered trademark of Dominic Mazzo. 1999.

3. Mullen T. NITRC CleanLine Tool/Resource Info. Available at https:// www.nitrc.org/projects/cleanline. 2012.

4. Fink L, Simola J, Tavano A, Lange E, Wallot S, Laeng B. From pre-processing to advanced dynamic modeling of pupil data. Behavior Research Methods. 2023.

5. Pfeffer T, Keitel C, Kluger DS, Keitel A, Russmann A, Thut G, et al. Coupling of pupil-and neuronal population dynamics reveals diverse influences of arousal on cortical processing. Elife. 2022;11:1–28.

6. Seropian L, Ferschneider M, Cholvy F, Micheyl C, Bidet-Caulet A, Moulin A. Comparing methods of analysis in pupillometry: application to the assessment of listening effort in hearing-impaired patients. Heliyon. 2022;8(6).

7. Ye R, Rua C, O’Callaghan C, Jones PS, Hezemans FH, Kaalund SS, et al. An in vivo probabilistic atlas of the human locus coeruleus at ultra-high field. Neuroimage [Internet]. 2021;225(October 2020):117487. Available from: https://doi.org/10.1016/j.neuroimage.2020.117487

8. Clewett D V., Lee TH, Greening S, Ponzio A, Margalit E, Mather M. Neuromelanin marks the spot: identifying a locus coeruleus biomarker of cognitive reserve in healthy aging. Neurobiol Aging [Internet]. 2016;37:117–26. Available from: http://dx.doi.org/10.1016/j.neurobiolaging.2015.09.019

9. Fernandes P, Regala J, Correia F, Gonçalves-Ferreira AJ. The human locus coeruleus 3-D stereotactic anatomy. Surgical and Radiologic Anatomy. 2012;34(10):879–85.

10. Betts MJ, Cardenas-blanco A, Kanowski M, Spottke A, Teipel SJ, Kilimann I, et al. Locus coeruleus MRI contrast is reduced in Alzheimer ’ s disease dementia and correlates with CSF A b levels. Alzheimer’s and Dementia: Diagnosis, Assessment and Disease Monitoring. 2019;11:281–5.

11. Corcoran AW, Alday PM, Schlesewsky M, Bornkessel-Schlesewsky I. Toward a reliable, automated method of individual alpha frequency (IAF) quantification. Psychophysiology. 2018;55(7).

12. Goljahani A, D’Avanzo C, Schiff S, Amodio P, Bisiacchi P, Sparacino G. A novel method for the determination of the EEG individual alpha frequency. Neuroimage [Internet]. 2012;60(1):774–86. Available from: http://dx.doi.org/10.1016/j.neuroimage.2011.12.001

13. Donoghue T, Haller M, Peterson EJ, Varma P, Sebastian P, Gao R, et al. Parameterizing neural power spectra into periodic and aperiodic components. Nat Neurosci [Internet]. 2020;23(12):1655–65. Available from: http://dx.doi.org/10.1038/s41593-020-00744-x

14. Gao R, Peterson EJ, Voytek B. Inferring synaptic excitation/inhibition balance from field potentials. Neuroimage [Internet]. 2017;158(July):70–8. Available from: http://dx.doi.org/10.1016/j.neuroimage.2017.06.078

15. Lu H, van der Linden D, Bakker AB. Changes in pupil dilation and P300 amplitude indicate the possible involvement of the locus coeruleus-norepinephrine (LC-NE) system in psychological flow. Sci Rep [Internet]. 2023;13(1):1–10. Available from: https://doi.org/10.1038/s41598-023-28781-z
